# Sex differences in the intergenerational link between maternal and neonatal whole blood DNA methylation: a genome-wide analysis in 2 birth cohorts

**DOI:** 10.1186/s13148-023-01442-8

**Published:** 2023-03-25

**Authors:** Jie Hu, Xin Xu, Jun Li, Yu Jiang, Xiumei Hong, Kathryn M. Rexrode, Guoying Wang, Frank B. Hu, Hongmei Zhang, Wilfried J. Karmaus, Xiaobin Wang, Liming Liang

**Affiliations:** 1 Division of Women’s Health, Department of Medicine, Bigham and Women’s Hospital and Harvard Medical School, Boston, MA USA; 2 Department of Epidemiology, Harvard T.H. Chan School of Public Health, 655 Huntington Avenue, Building 2, Room 207, Boston, MA 02115 USA; 3 Department of Nutrition, Harvard T.H. Chan School of Public Health, Boston, MA USA; 4 Division of Epidemiology, Biostatistics, & Environmental Health, School of Public Health, University of Memphis, Memphis, TN USA; 5 Center On the Early Life Origins of Disease, Department of Population, Family and Reproductive Health, Johns Hopkins University Bloomberg School of Public Health, Baltimore, MD USA; 6 Channing Division of Network Medicine, Department of Medicine, Brigham and Women’s Hospital and Harvard Medical School, Boston, MA USA; 7 Department of Pediatrics, Johns Hopkins University School of Medicine, Baltimore, MD USA; 8 Department of Biostatistics, Harvard T.H. Chan School of Public Health, Boston, MA USA

**Keywords:** DNA methylation, Intergenerational transmission, Sex difference, Developmental origin of disease, Birth cohort

## Abstract

**Background:**

The mother–child inheritance of DNA methylation (DNAm) variations could contribute to the inheritance of disease susceptibility across generations. However, no study has investigated patterns of mother–child associations in DNAm at the genome-wide scale. It remains unknown whether there are sex differences in mother–child DNAm associations.

**Results:**

Using genome-wide DNAm profiling data (721,331 DNAm sites, including 704,552 on autosomes and 16,779 on the X chromosome) of 396 mother–newborn pairs (54.5% male) from the Boston Birth Cohort, we found significant sex differences in mother–newborn correlations in genome-wide DNAm patterns (Spearman’s *rho* = 0.91–0.98; *p* = 4.0 × 10^–8^), with female newborns having stronger correlations. Sex differences in correlations were attenuated but remained significant after excluding X-chromosomal DNAm sites (Spearman’s *rho* = 0.91–0.98; *p* = 0.035). Moreover, 89,267 DNAm sites (12.4% of all analyzed, including 88,051 [12.5% of analyzed] autosomal and 1,216 [7.2% of analyzed] X-chromosomal sites) showed significant mother–newborn associations in methylation levels, and the top autosomal DNAm sites had high heritability than the genome-wide background (e.g., the top 100 autosomal DNAm sites had a medium *h*^*2*^ of 0.92). Additionally, significant interactions between newborn sex and methylation levels were observed for 11 X-chromosomal and 4 autosomal DNAm sites that were mapped to genes that have been associated with sex-specific disease/traits or early development (e.g., *EFHC2*, *NXY*, *ADCYAP1R1*, and *BMP4*). Finally, 18,769 DNAm sites (14,482 [77.2%] on the X chromosome) showed mother–newborn differences in methylation levels that were significantly associated with newborn sex, and the top autosomal DNAm sites had relatively small heritability (e.g., the top 100 autosomal DNAm sites had a medium *h*^*2*^ of 0.23). These DNAm sites were mapped to 2,532 autosomal genes and 978 X-chromosomal genes with significant enrichment in pathways involved in neurodegenerative and psychological diseases, development, neurophysiological process, immune response, and sex-specific cancers. Replication analysis in the Isle of Wight birth cohort yielded consistent results.

**Conclusion:**

In two independent birth cohorts, we demonstrated strong mother–newborn correlations in whole blood DNAm on both autosomes and ChrX, and such correlations vary substantially by sex. Future studies are needed to examine to what extent our findings contribute to developmental origins of pediatric and adult diseases with well-observed sex differences.

**Supplementary Information:**

The online version contains supplementary material available at 10.1186/s13148-023-01442-8.

## Background

DNA methylation (DNAm) is a major epigenetic mechanism that regulates gene expression, genomic imprinting, and chromosomal stability without altering the genomic sequences [1, 2]. The most mechanistically understood DNAm is on the C5 position of a cytosine–phosphate–guanine (CpG) dinucleotide [3]. About 60–80% of CpGs in the human genome are generally methylated [4]. From a life course perspective, the intrauterine period is critical for the establishment of DNAm. Most parental DNAm marks are removed shortly after fertilization through active (paternal genome) or passive (maternal genome) demethylation and then re-established upon implantation through de novo methylation [5, 6]. In addition, DNAm levels are affected by both genetic and exogenous factors [7, 8], and are particularly sensitive to prenatal environmental perturbations, such as exposure to environmental toxins [9–12], maternal smoking [13–15], substance use [16], nutritional factors [17], and psychological conditions [18]. Moreover, such changes in the DNAm patterns related to early-life environmental exposure could have long-term impacts on disease susceptibilities [19, 20]. In a number of existing studies, DNAm variations in leukocytes (i.e., white blood cells) have been associated with the development of various human diseases [2, 21].

The mother could affect her fetus’ DNAm via both genetic and environmental factors, including her external environment and in utero environment. A few candidate gene-based studies have demonstrated the intergenerational and transgenerational transmission of DNAm status of certain cancer-related genes in humans, which might contribute to the inheritance of cancer susceptibility across generations [22, 23]. Additionally, a few genome-wide DNAm studies have examined contributions of genetic variations and environmental factors to transgenerational inheritance of genome-wide methylation status, indicating that most transgenerational similarity in DNAm is attributable to genetics [24, 25]. A recent study also identified shared genetic variants for DNAm levels and complex diseases [26]. However, no study, to our knowledge, has examined the intergenerational similarity of DNAm patterns between mother and newborns at the genome-wide level, which could reveal the mechanisms underlying the developmental origins of many human diseases.

Genetically, males and females differ in their sex chromosomes. Remarkable sex differences in both pediatric and adult diseases have been observed, but the underlying mechanisms are poorly understood. In humans, several thousands of DNAm sites, enriched on both CpG island shores and imprinted genes, are methylated differently by sex [25, 27–29]. Sex differences in DNAm patterns could contribute to sex-biased epigenetic regulation of gene expression and further contribute to sexually differentiated traits [30, 31]. However, it remains unknown whether the maternal–neonatal transmission of DNAm patterns differ by sex, and whether such differences contribute to sex differences in development and diseases.

In this study, we aimed to address the above knowledge gaps by investigating mother–newborn correlations in DNAm by newborn sex for all methylation sites, as well as for autosomal and X-chromosomal sites separately. We further examined differences in methylation levels for individual DNAm sites between mothers and their newborns (ΔDNAm) and tested for their associations with newborn sex. We used data of mother–newborn dyads from the Boston Birth Cohort (BBC), a predominantly urban, low-income minority birth cohort in the USA, and the Isle of Wight birth cohort (IOWBC) from the UK (as a replication cohort). Genome-wide DNAm profiling was performed on maternal peripheral blood and neonatal cord blood samples using the Illumina Infinium MethylationEPIC (BBC and part of IOWBC) and the HumanMethylation450 (part of IOWBC) BeadChips.

## Results

This study included 396 mother–newborn pairs from the BBC; 180 (45.5%) newborns were female and 216 (54.5%) were male. Most newborns were delivered through natural birth (131 [72.8%] female and 144 [66.7%] male newborns); female newborns were less likely to be born preterm when compared to male newborns (female vs. male: 20 [11.1%] vs. 45 [20.8%]; *p* = 0.01). About half of the newborns in this study were the first birth. Most mothers included in this study were non-smokers during pregnancy and self-identified as Black. Additionally, maternal age at delivery ranged from 15.3 to 46.8 years. As replication sets, this study included 48 pre-pregnancy mother–newborn pairs (maternal blood sample collected at the age of 18 years; 18 female and 30 male newborns) and 93 early-pregnancy mother–newborn pairs (maternal blood sample collected in 10–21 weeks of gestation; 47 female and 46 male newborns) from the IOWBC. Compared to the BBC, mothers from the IOWBC were all White, were younger at delivery (ranged from 21 to 33 years), had lower percentages of C-section delivery and preterm delivery, and had higher percentages of smoking during pregnancy. Notably, except for preterm delivery in BBC, all other maternal characteristics did not differ by newborn sex in both BBC and IOWBC (Table 1).

**Table 1:** Characteristics of mother-newborn pairs by newborn sex in the BBC and IOWBC

	Mother-newborn pairs from the BBC			Pre-pregnancy mother-newborn pairs from the IOWBC			Early-pregnancy mother-newborn pairs from the IOWBC		
	Female	Male	*P*	Female	Male	*P*	Female	Male	*P*
Total N	180	216		18	30		47	46	
Maternal age at delivery, yr*	27.5 (15.3—46.2)	27.6 (16.5—46.8)	0.91	23.0 (20.3—26.6)	23.4 (21.3—27.3)	0.91	23.3 (21.2—29.7)	23.3 (21.2—33.3)	0.93
Type of delivery			0.23			0.78			0.97
Spontaneous vaginal	131 (72.8%)	144 (66.7%)		15 (83.3%)	24 (80.0%)		40 (85.1%)	39 (84.8%)	
C-section	49 (27.2%)	72 (33.3%)		3 (16.7%)	6 (20.0%)		7 (14.9%)	7 (15.2%)	
Preterm delivery			0.01			0.88			0.57
No	160 (88.9%)	171 (79.2%)		17 (94.4%)	28 (93.3%)		45 (95.7%)	45 (97.8%)	
Yes	20 (11.1%)	45 (20.8%)		1 (5.6%)	2 (6.7%)		2 (4.3%)	1 (2.2%)	
Maternal smoking during pregnancy	0.87			0.95			0.57		
No	159 (88.3%)	193 (89.4%)		11 (61.1%)	18 (60.0%)		29 (61.7%)	31 (67.39%)	
Yes	21 (11.7%)	23 (10.6%)		7 (38.9%)	12 (40.0%)		18 (38.3%)	15 (32.6%)	
Maternal race/ethnicity			0.57			–			–
Black	115 (63.9%)	145 (67.1%)		–	–		–	–	
Non-Black	65 (36.1%)	71 (32.9%)		–	–		–	–	
Birth order			0.23			0.65			0.44
First birth	75 (41.7%)	108 (50.0%)		12 (66.7%)	18 (60.0%)		23 (48.9%)	25 (54.4%)	
Second birth	52 (28.9%)	50 (23.1%)		4 (22.2%)	8 (26.7%)		11 (36.2%)	13 (28.3%)	
Third birth and later	53 (29.4%)	58 (26.9%)		2 (11.1%)	4 (13.3%)		7 (14.9%)	8 (17.4%)	

^*^Median (range) of maternal age at delivery

A *p*-value < 0.05 is defined as statistical significant

After quality control steps, 721,331 DNAm sites were available for data analyses in the BBC, with 704,552 on autosomes and 16,779 on the X chromosome. In the IOWBC, 218,259 DNAm sites (including 208,573 autosomal and 9,686 X-chromosomal DNAm sites) for pre-pregnancy mother–newborn pairs and 401,539 DNAm sites (including 391,902 autosomal and 9,637 X-chromosomal DNAm sites) for early-pregnancy mother–newborn pairs were available for data analysis.

### Sex differences in sample-wise correlations between maternal and neonatal DNAm patterns

**Fig. 1:**
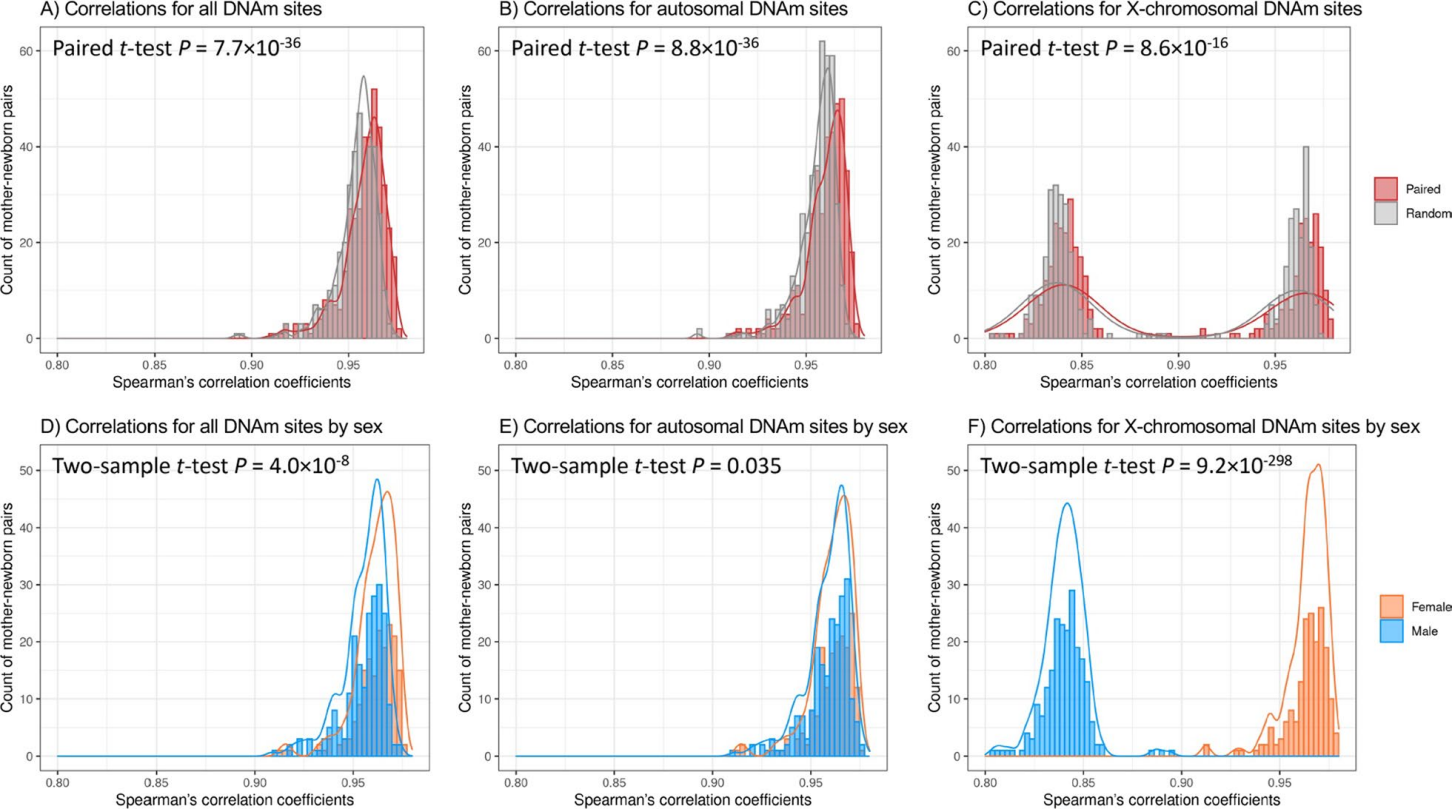
Distributions of overall maternal–newborn correlations in DNAm in the BBC. Overall mother–newborn correlations A-C) comparing paired maternal–newborn samples (marked as red) and randomly paired maternal–newborn samples (computed from 10 permutation; marked as gray) and D-F) comparing between newborn sex for all, autosomal, and X-chromosomal DNAm sites

The Spearman’s correlation coefficients of the 396 mother–newborn pairs distributed significantly different than those of randomly selected pairs of unrelated mothers and newborns (paired *t* test *p* = 7.7 × 10^–36^), and correlation coefficients of matched mother–newborn pairs were significantly stronger than that of unrelated pairs for both autosomal and X-chromosomal DNAm sites (Fig. 1 A-C). Similar results were observed in mother–male and mother–female pairs (Additional file 1: Figure S1), indicating that the observed mother–newborn correlation in DNAm patterns was mostly driven by family relatedness. In addition, the maternal-neonatal correlations in DNAm patterns significantly differed by newborn sex (*p* = 4.0 × 10^–8^), with female newborns having relatively stronger correlations (Fig. 1D). For autosomal DNAm sites, the distributions of correlation coefficients were very similar between sexes, but female newborns showed higher maternal-neonatal correlation (*p* = 0.035) (Fig. 1E). For X-chromosomal DNAm sites, female newborns had larger correlation coefficients than male newborns (*rho*_male_ = 0.80–0.89; *rho*_female_ = 0.91–0.98; two-sample *t* test *p* = 9.2 × 10^–298^) (Fig. 1F). In replication analyses in the IOWBC, female newborns also showed significantly higher overall correlations, especially for X-chromosomal DNAm sites (two-sample *t* test *p* = 1.5 × 10^–28^ for early-pregnancy pairs and *p* = 2.6 × 10^–42^ for early-pregnancy pairs) (Additional file 1: Figure S2).

### Sex differences in site-wise associations between maternal and neonatal methylation levels

**Fig. 2:**
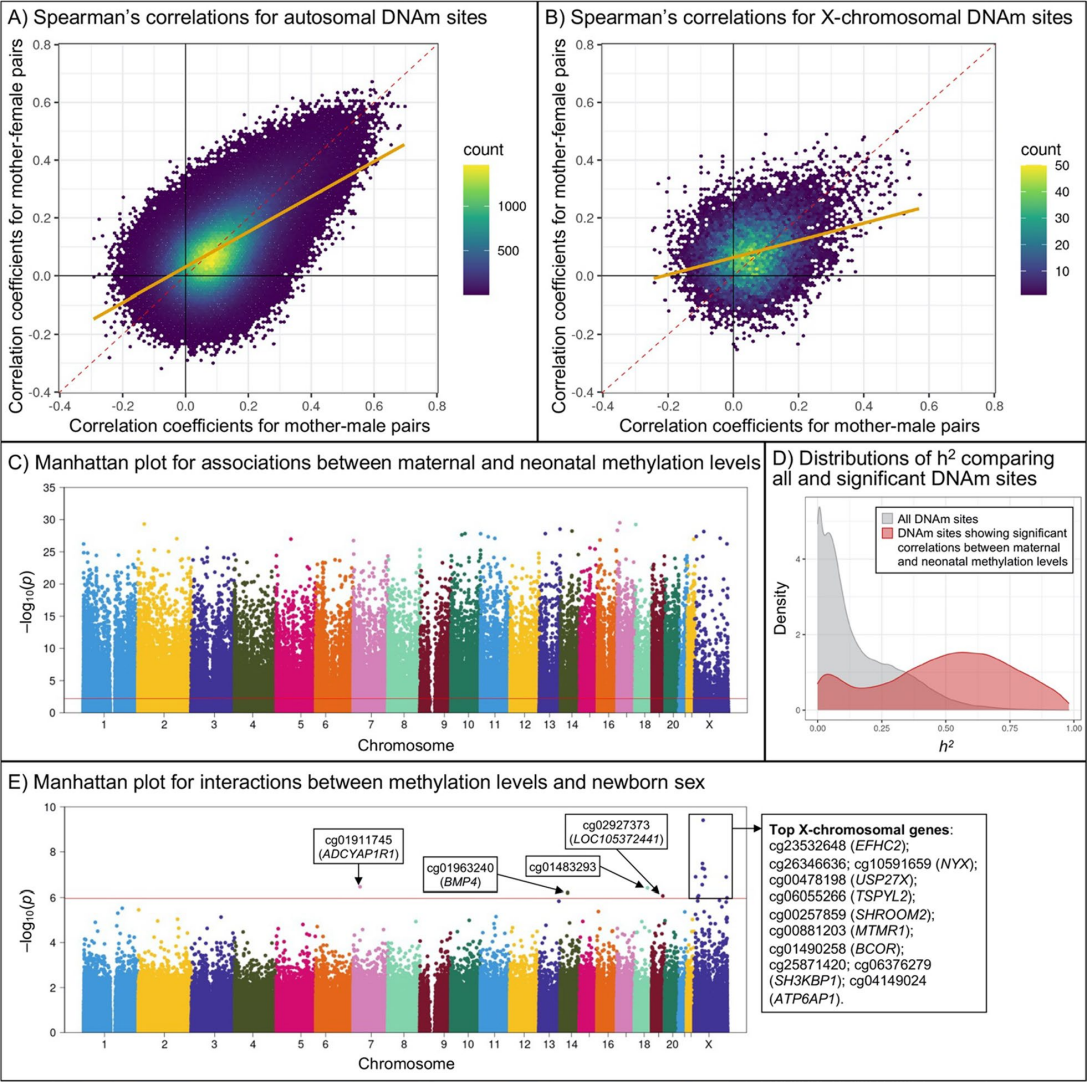
Sex differences in mother–newborn correlations in methylation levels at individual DNAm sites. We first illustrated Spearman correlations in mother–newborn correlations in methylation levels of individual DNAm sites on A) autosomes and B) the X chromosome, stratified by newborn sex. The orange regression lines represent the concordance of correlation coefficients for mother–male and mother–female pairs. Second, we used C) Manhattan plot to show the distribution of p values from likelihood ratio test that estimated the associations in methylation levels at individual DNAm sites between mothers and their newborns; the red horizontal line marks FDR = 0.05. We also used D) density plot to compare the heritability of DNAm sites showing significant mother–newborn associations to that of DNAm sites across the whole genome using data from a published study on DNAm quantitative trait loci [25], but only the heritability of autosomal DNAm sites was available. Finally, we used E) Manhattan plot to show the distribution of p values of the interaction term of newborn sex and maternal methylation levels (neonatal methylation levels as the outcome) for individual DNAm sites; the red horizontal line marks FDR = 0.05. Linear regression models were adjusted for maternal age at delivery, maternal race/ethnicity, maternal smoking, preterm birth, type of delivery, and 36 surrogate variables. DNAm, DNA methylation; *h*^*2*^, heritability

We first compared sex differences in Spearman’s correlation coefficients for individual DNAm sites between mothers and their newborns, plotting the mother–newborn correlation coefficients by newborn sex (Fig. 2 A-B). The correlation coefficients for most autosomal and X-chromosomal DNAm sites distributed close to the diagonal, indicating that the correlation coefficients were similar between mother–male and mother–female pairs. But the regression lines for both autosomal and X-chromosomal DNAm sites sloped to the horizontal axis, indicating that the correlations for mother–male pairs were slightly higher than those for mother–female pairs across the whole genome, and even higher for X-chromosomal DNAm sites (Fig. 2 A-B).

We observed 89,267 DNAm sites with significant associations between maternal and neonatal methylation levels (false discovery rate [FDR] < 0.05 in the likelihood ratio test); 88,051 of these DNAm sites were on autosomes (12.5% of all analyzed autosomal DNAm sites) and 1,216 were on the X chromosome (7.2% of all analyzed X-chromosomal DNAm sites) (Fig. 2C and Additional file 2: Table S1). Based on the genetic heritability (*h*^*2*^) of DNAm levels from a published study on DNAm quantitative trait loci (mQTL) [25], we observed that autosomal DNAm sites showing significant mother–newborn associations in our study had relatively higher *h*^*2*^ when compared to the genome-wide DNAm sites (Fig. 2D). Moreover, DNAm sites with the highest *h*^*2*^ were also the top autosomal DNAm sites showing significant mother–newborn associations in methylation levels (e.g., the median *h*^*2*^ of top 100 autosomal DNAm sites was 0.92) (Additional file 1: Figure S3 and S4). Finally, the identified 89,267 DNAm sites were mapped to 18,186 genes; 15,871 of these genes were available in the GSE27272 dataset [32], and 8,572 (54.0%) genes also show a significant correlation in maternal–newborn gene expression (FDR < 0.05 for 15,871 comparisons; Additional file 2: Table S2).

In sex interaction analyses, 15 DNAm sites (4 on autosomes; 11 on the X chromosome) showed significant interactions between maternal methylation levels and newborn sex with an FDR < 0.05 (Fig. 2E and Table 2). The 4 autosomal DNAm sites were mapped to 3 genes, including cg01911745 on *ADCYAP1R1*, which has been related to post-traumatic stress disorder, accommodative spasm, and childhood asthma [33]; cg01963240 on *BMP4*, which regulates embryonic development and adipogenesis and may also be involved in the pathology of multiple cardiovascular diseases and human cancers [34]; and cg02927373 on *LOC105372441*, which encodes a long noncoding RNA. Only 1 autosomal DNAm site (cg01963240 on *BMP4*) was available in the IOWBC, which showed significant mother–newborn correlations in likelihood ratio test (*p* = 2.6 × 10^–42^) in pre-pregnancy pairs, but did not show significant sex interactions in both pre-pregnancy and early-pregnancy pairs. The 11 X-chromosomal DNAm sites were mapped to 9 genes that were related to sex-specific disease/traits or early development, including cg23532648 on *EFHC2* (development of epilepsy [35] and social cognitive abilities [36]), cg10591659 on *NYX* (X-linked congenital stationary night blindness [37, 38]), cg00478198 on *USP27X* (X-linked cognitive disability [39]), cg06055266 on *TSPYL2* (encodes a member of the testis-specific protein that may play a role in the suppression of tumor growth [40]), cg00257859 on *SHROOM2* (male-specific autism spectrum disorder [41]), cg00881203 on *MTMR1* (plays a role in muscle formation during fetal development [42]), cg01490258 on *BCOR* (encodes a protein that acts as an interacting corepressor of BCL6, and its mutations have been related to multiple tumors [43]), cg06376279 on *SH3KBP1* (plays an essential role in the stimulation of B cell activation [44]), and cg04149024 on *ATP6AP1* (the deficiency of the encoded protein has been related to immunodeficiency with hepatopathy, cognitive impairment, and abnormal protein glycosylation [45]). In the IOWBC, 8 of the 11 X-chromosomal DNAm sites (6 genes) were available, and 1 DNAm site (cg10591659 on *NYX*) showed significant sex interactions (p-for-interaction < 0.05) in both pre-pregnancy and early-pregnancy pairs. Finally, because birth order could potentially impact fetal DNAm profile, we performed a sensitivity analysis additionally adjusting for parity, and the results remained unchanged (Additional file 1: Figure S5).

**Table 2:** DNAm sites with significant interaction between maternal methylation and newborn sex on neonatal methylation in the BBC and IOWBC

DNAm site	Chr	BP	Mother–newborn pairs from the BBC						IOWBC – early-pregnancy pairs				IOWBC – pre-pregnancy pairs				Mapped gene	Functional annotation	Relation to CpG Island
			Mother–newborn association		The interaction term of newborn sex and maternal DNAm level				Mother–newborn association *P*	The interaction term of newborn sex and maternal DNAm level			Mother–newborn association *P*	The interaction term of newborn sex and maternal DNAm level					
			*P*	FDR	Beta	s.e	*P*	FDR		Beta	s.e	*P*		Beta	s.e	*P*			
cg23532648	X	44,169,550	7.2 × 10^–29^	7.4 × 10^–24^	0.515	0.080	3.9 × 10^–10^	0.0003	0.001	0.704	0.655	0.30	0.002	0.459	0.282	0.11	*EFHC2*	Body	Island
cg26346636	X	41,301,703	6.1 × 10^–7^	1.8 × 10^–5^	− 0.479	0.085	3.3 × 10^–8^	0.0104	0.01	0.195	0.491	0.70	0.0002	− 0.068	0.200	0.74			Island
cg10591659	X	41,306,768	1.5 × 10^–18^	1.2 × 10^–15^	0.454	0.082	5.3 × 10^–8^	0.0104	5.6 × 10^–6^	− 1.210	0.431	0.02	0.001	− 0.647	0.291	0.03	*NYX*	5'UTR/1stExon	
cg00478198	X	49,644,500	2.7 × 10^–6^	6.9 × 10^–5^	− 0.283	0.051	5.8 × 10^–8^	0.0104	0.02	− 0.283	0.333	0.41	0.99	0.009	0.221	0.97	*LOC158572 USP27X*	TSS1500 5'UTR/1stExon	Island
cg06055266	X	53,111,187	4.4 × 10^–8^	1.8 × 10^–6^	− 0.275	0.051	1.3 × 10^–7^	0.0135	0.32	0.258	0.321	0.44	0.004	0.278	0.140	0.05	*TSPYL2*	TSS1500	N_Shore
cg00257859	X	9,815,644	4.1 × 10^–28^	2.4 × 10^–23^	0.404	0.075	1.3 × 10^–7^	0.0135									*SHROOM2*	Body	
cg00881203	X	149,930,926	1.4 × 10^–14^	2.9 × 10^–12^	− 0.354	0.066	1.3 × 10^–7^	0.0135									*MTMR1*	Body	S_Shore
cg01490258	X	40,030,456	1.8 × 10^–6^	4.7 × 10^–5^	− 0.395	0.075	2.9 × 10^–7^	0.0260	0.14	− 0.635	0.788	0.44	0.70	− 0.037	0.173	0.83	*BCOR*	5'UTR	N_Shore
cg01911745	7	31,091,055	1.6 × 10^–7^	5.6 × 10^–6^	− 0.396	0.076	3.6 × 10^–7^	0.0287									*ADCYAP1R1*	TSS1500	N_Shore
cg01483293	18	59,251,006	2.8 × 10^–5^	0.0005	− 0.474	0.092	4.0 × 10^–7^	0.0289											
cg01963240	14	54,421,117	8.4 × 10^–6^	0.0002	− 0.514	0.101	6.3 × 10^–7^	0.0413	3.2 × 10^–14^	− 0.716	0.387	0.09	0.18	0.274	0.308	0.38	*BMP4*	5'UTR	Island
cg25871420	X	23,348,969	9.5 × 10^–15^	2.1 × 10^–12^	0.380	0.076	8.3 × 10^–7^	0.0469	1.1 × 10^–5^	0.890	0.592	0.16	0.12	0.337	0.268	0.22			N_Shore
cg02927373	19	51,347,703	5.9 × 10^–5^	0.001	0.476	0.095	8.4 × 10^–7^	0.0469									*LOC105372441*	Body	
cg06376279	X	19,585,128	1.3 × 10^–19^	1.5 × 10^–16^	0.423	0.085	9.5 × 10^–7^	0.0488									*SH3KBP1*	Body	
cg04149024	X	153,656,891	8.0 × 10^–6^	0.0002	− 0.301	0.060	1.0 × 10^–6^	0.0493	0.11	0.438	0.445	0.35	0.57	0.029	0.173	0.87	*ATP6AP1*	TSS200	Island

Chr, chromosome; BP, position; FDR, false discovery rate; CpG, cytosine–phosphate–guanine dinucleotide; s.e., standard error

### Differences in maternal and neonatal methylation levels and associations with newborn sex

**Fig. 3:**
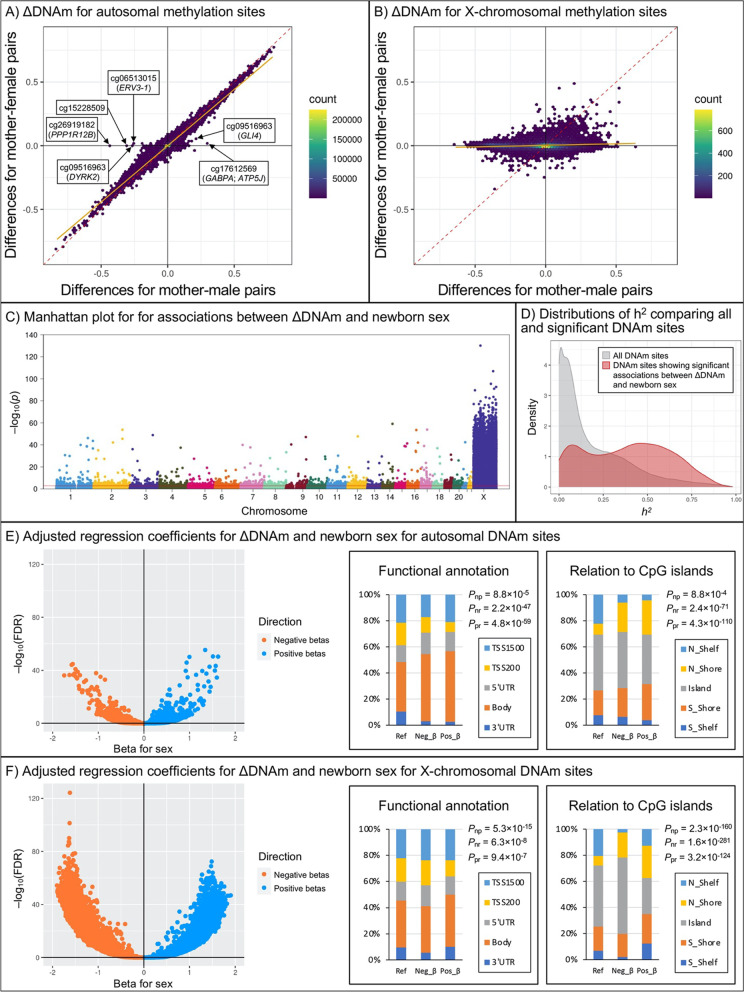
Sex differences in associations between differences in maternal-neonatal methylation levels (ΔDNAm) and newborn sex at individual DNAm sites. We first illustrated differences in maternal–neonatal methylation levels of individual DNAm sites (ΔDNAm) on A) autosomes and B) the X chromosome, stratified by newborn sex. The orange regression lines represent the concordance of ΔDNAm for mother–male and mother–female pairs. Then, we used C) Manhattan plot to show the distribution of p values of associations between ΔDNAm and newborn sex; the red horizontal line marks FDR = 0.05. We also used D) density plot to compare the heritability of DNAm sites showing significant associations between ΔDNAm and newborn sex to that of DNAm sites across the whole genome using data from a published study on DNAm quantitative trait loci [25], but only the heritability of autosomal DNAm sites was available. Finally, we illustrated the regression coefficients for E) autosomal and F) X-chromosomal DNA sites; blue dots represent positive betas (mother–male differences larger than mother–female differences), while orange dots represent negative betas (mother–male differences smaller than mother–female differences). We also presented the percentages of function annotation and the relation to CpG island for individual E) autosomal and F) X-chromosomal DNA sites, and compared the distributions between genome-wide references, negative betas, and positive betas using chi-square test. Linear regression models were adjusted for maternal age at delivery, maternal race/ethnicity, maternal smoking, preterm birth, type of delivery, and 37 surrogate variables. DNAm, DNA methylation; ΔDNAm, differences in maternal-neonatal methylation levels; *h*^*2*^, heritability; Ref, the distributions of mapped genes of all DNAm sites that were included in the analysis; Neg_β, the distributions of mapped genes of DNAm sites that showed significant negative associations between ΔDNAm and newborn sex; Pos_β, the distributions of mapped genes of DNAm sites that showed significant positive associations between ΔDNAm and newborn sex; *P*_np_, *P*_nr_, and *P*_pr_ represent p-values comparing the distributions of functional annotations for negative *β*s vs. positive *β*s, negative *β*s vs. references, and positive *β*s vs. references, respectively

We first calculated differences between maternal and neonatal methylation levels for each DNAm site (ΔDNAm). For autosomal DNAm sites, the mean (standard deviation) of ΔDNAm was 0.003 (0.071), suggesting that maternal and neonatal methylation levels of individual DNAm sites were very similar. When plotting the autosomal ΔDNAm by newborn sex (Fig. 3A), we observed that the majority of DNAm sites were distributed next to the origin, but there were a few DNAm sites that showed large mother–male differences. These DNAm sites were mapped to genes that play mechanistic roles in multiple cancers, such as cg06513015 on *ERV3-1*, cg09516963 on *DYRK2*, cg26919182 on *PPP1R12B*, and cg17612569 on *GABPA* and *ATP5J* [46–50]. For X-chromosomal DNAm sites, the means (standard deviations) of ΔDNAm were -0.058 (0.191) for mother–male pairs and 0.002 (0.065) for mother–female pairs, indicating that X-chromosomal DNAm sites in male newborns were generally less methylated than their mothers. When plotting the X-chromosomal ΔDNAm by newborn sex, most DNAm sites were distributed close to the horizontal axis, suggesting that methylation levels of X-chromosomal DNAm sites were very similar between mothers and their female newborns but showed large differences between mothers and their male newborns (Fig. 3B).

In linear regression analysis, we coded newborn sex as 0 = female and 1 = male. Therefore, a positive *β* (regression coefficient for sex > 0) represents that the ΔDNAm for a certain DNAm site in mother–male pairs was larger than that in mother–female pairs. After adjusting for covariates, 18,769 DNAm sites showed significant associations between ΔDNAm and newborn sex (FDR < 0.05) (Fig. 3C); 4,287 of these DNAm sites were on autosomes (0.6% of all analyzed autosomal DNAm sites) and 14,482 were on the X chromosome (86.3% of all analyzed X-chromosomal DNAm sites) (Additional file 2: Table S3). Notably, 1,351 of these identified autosomes DNAm sites showed significant sex differences in methylation levels in cord blood samples (Additional file 2: Table S4) based on a recent study from the Pregnancy And Childhood Epigenetics (PACE) consortium [51]. Additionally, autosomal DNAm sites showing significant associations between ΔDNAm and newborn sex also had relatively higher *h*^*2*^ than the genome-wide background (Fig. 3D). But the top DNAm sites showing significant and strong associations between ΔDNAm and newborn sex had relatively smaller *h*^*2*^ (e.g., the top 100 autosomal DNAm sites had a medium *h*^*2*^ of 0.23), and DNAm sites with the highest heritability (*h*^*2*^ ≥ 0.95) were not showing significant associations between ΔDNAm and newborn sex (FDR > 0.1) (Additional file 1: Figure S6). As a replication, we estimated associations between ΔDNAm and newborn sex based on overlapping DNAm sites in the IOWBC. For all identified 18,769 DNAm sites, 8,896 (7,616 on the X chromosome) were available in the pre-pregnancy pairs; 1,245 (1,196 on the X chromosome) of these DNAm sites showed significant associations between ΔDNAm and newborn sex, and the direction of associations for 1,114 (89.5%) of these DNAm sites was consistent with the BBC (*p* = 6.7 × 10^–147^ in chi-square test for concordance) (Additional file 1: Figure S7). Similarly, in the early-pregnancy pairs from the IOWBC, where 9,986 (7,578 on the X chromosome) of all identified 18,769 DNAm sites were available, 1,827 DNAm sites (1,739 on the X chromosome) showed significant associations between ΔDNAm and newborn sex, and the direction of associations for 1,664 (91.1%) of these DNAm sites were consistent with the BBC (*p* = 4.8 × 10^–248^ in chi-square test for concordance) (Additional file 1: Figure S8).

For the 4,287 autosomal DNAm sites showing significant associations between ΔDNAm and newborn sex, 2,933 of them (1,419 [48.4%] with negative *β*s and 1,514 [51.6%] with positive *β*s) were mapped to 2,532 genes. More than 50% of these autosomal DNAm sites were located on gene bodies, while about 30% of them were located at or upstream of the transcriptional start site (TSS); about 90% of these autosomal DNAm sites were in the CpG islands or CpG island shores. For these identified autosomal DNAm sites, the distributions of their functional annotations and relation to CpG islands were significantly different when comparing to the genomic references (*p* < 5 × 10^–59^), and significantly differed by the directions of *β*s (p_neg_vs_pos_ = 8.8 × 10^–5^ for functional annotations and p_neg_vs_pos_ = 8.8 × 10^–4^ for relation to CpG islands) (Fig. 3E). The top autosomal DNAm sites were mapped to genes that have been associated with breast cancer and other malignancies (e.g., cg02325951 on *FOXN3*, cg20262915 and cg19765154 on *NAB1*, and cg07850329 on *BAG1*) [52–54], as well as childhood neurodegeneration (e.g., cg12607525 on *UBTF*) [55] (Additional file 2: Table S3). A few top autosomal DNAm sites also showed sex-differed methylation in newborns (e.g., cg02325951, cg04946709, and cg17612569) [56].

For the 14,482 X-chromosomal DNAm sites that showed significant associations between ΔDNAm and newborn sex, 10,994 of them (6,843 [62.2%] with negative *β*s and with 4,151 [37.8%] positive *β*s) were mapped to 978 genes. Most of the identified X-chromosomal DNAm sites were on gene bodies (35.7% of those with negative *β*s vs. 39.9% of those with positive *β*s) or upstream of the TSS (42.8% of those with negative *β*s vs. 36.2% of those with positive *β*s), and the distributions of these functional annotations for negative *β*s and positive *β*s were significantly different (p_neg_vs_pos_ = 5.3 × 10^–15^). More DNAm sites with negative *β*s (95.6%) than those with positive *β*s (74.9%) were located in CpG islands or CpG island shores (p_neg_vs_pos_ = 2.3 × 10^–160^). For these X-chromosomal DNAm sites, the distributions of their functional annotations (p ≤ 9.4 × 10^–7^) and relation to CpG islands (p ≤ 3.2 × 10^–124^) were significantly different from the genomic references (Fig. 3F). Top X-chromosomal DNAm sites were mapped to several genes that are involved in human tumorigenesis (e.g., cg24741068 on *RBM3*, cg26565914 on *LINC00629*, cg27076487 on *LDOC1*, cg00209850 on *ACSL4*, cg03831206 on *OTUD5*, and cg06042004 on *LAGE3*) [57–62] and X-linked cognitive/intellectual disability (e.g., cg15661671 on *UBE2A*, cg15774752 on *ZNF711*, cg12481479 on *TSPAN7*, and cg05785344 on *BCORL1*) [63–66] (Additional file 2: Table S3).

In pathway enrichment analysis, we included all the identified genes associated with newborn sex (2,532 genes on autosomes and 978 genes on the X chromosome) and estimated their enrichment in biological pathways and diseases. We observed significant enrichment in 58 biological pathways (FDR < 0.005); the top pathways were involved in biological processes for neurodegenerative and psychological diseases (Alzheimer disease, Parkinson's disease, bipolar disorder, etc.), as well as biological pathways related to development, neurophysiological process, immune response, and sex-specific cancers (e.g., prostate cancer) (Table 3 and Additional file 2: Table S5). In addition, these genes were also enriched in multiple diseases; top diseases included thoracic diseases and cancers (e.g., lung and respiratory tract diseases and neoplasms), sex-specific diseases (e.g., male and female urogenital diseases, female genital diseases and neoplasms, and breast diseases and neoplasms), neurodevelopmental disorders (e.g., intellectual disability, neurobehavioral manifestations, X-linked mental retardation), and several X-linked genetic diseases (Additional file 2: Table S6).

**Table 3:** Top enriched pathways (FDR_enrichment_ < 0.005) for DNAm sites with significant associations between maternal-neonatal differences in methylation levels (ΔDNAm) and newborn sex

Enriched pathway	Number of genes in pathway map	Number of genes in data	Enrichment analysis	
			*P*	FDR
Protein folding and maturation_POMC processing	30	19	4.2 × 10^–13^	6.1 × 10^–10^
Tau pathology in Alzheimer disease	55	21	6.6 × 10^–9^	3.4 × 10^–6^
LRRK2 in neurons in Parkinson's disease	33	16	6.8 × 10^–9^	3.4 × 10^–6^
Development_Positive regulation of WNT/Beta-catenin signaling in the nucleus	69	22	1.3 × 10^–7^	4.7 × 10^–5^
Development_Positive regulation of WNT/Beta-catenin signaling in the cytoplasm	76	23	2.0 × 10^–7^	5.8 × 10^–5^
Neurophysiological process_Constitutive and activity-dependent synaptic AMPA receptor delivery	59	19	7.8 × 10^–7^	0.0002
Development_Hedgehog signaling	94	25	9.0 × 10^–7^	0.0002
Immune response_IL-3 signaling via ERK and PI3K	102	26	1.3 × 10^–6^	0.0002
Ligand-independent activation of Androgen receptor in Prostate Cancer	67	20	1.5 × 10^–6^	0.0002
Signal transduction_PKA signaling	51	17	1.7 × 10^–6^	0.0002
Regulation of GSK3 beta in bipolar disorder	46	16	1.8 × 10^–6^	0.0002
Cytoskeleton remodeling_Neurofilaments in axon growth and synapses	23	11	2.0 × 10^–6^	0.0002
Transcription_Negative regulation of HIF1A function	69	20	2.6 × 10^–6^	0.0003
Cytoskeleton remodeling_Regulation of actin cytoskeleton organization by the kinase effectors of Rho GTPases	58	18	2.8 × 10^–6^	0.0003
Neurophysiological process_Constitutive and regulated NMDA receptor trafficking	65	19	4.0 × 10^–6^	0.0004
Signal transduction_Adenosine A2B receptor signaling pathway	71	20	4.2 × 10^–6^	0.0004
Development_WNT/Beta-catenin signaling in the cytoplasm	55	17	5.5 × 10^–6^	0.0005
Role of tumor-infiltrating B cells in anti-tumor immunity	91	23	6.2 × 10^–6^	0.0005
Development_Negative regulation of WNT/Beta-catenin signaling at the receptor level	45	15	7.0 × 10^–6^	0.0005
Neurogenesis_NGF/ TrkA MAPK-mediated signaling	105	25	7.9 × 10^–6^	0.0006
Immune response_Fc epsilon RI pathway: calcium-dependent signaling	68	19	8.3 × 10^–6^	0.0006
Signal transduction_Angiotensin II signaling via Beta-arrestin	57	17	9.5 × 10^–6^	0.0006
Neurophysiological process_Synaptic vesicle fusion and recycling in nerve terminals	52	16	1.1 × 10^–5^	0.0007
Nociception_Nociceptin receptor signaling	76	20	1.3 × 10^–5^	0.0008
Development_Negative feedback regulation of WNT/Beta-catenin signaling	37	13	1.5 × 10^–5^	0.0009
Chemotaxis_Lysophosphatidic acid signaling via GPCRs	129	28	1.5 × 10^–5^	0.0009
Development_WNT/Beta-catenin signaling pathway. Signalosome	43	14	1.9 × 10^–5^	0.0010
Development_PTHR1 in bone and cartilage development	78	20	2.0 × 10^–5^	0.0010
Immune response_Platelet activating factor/ PTAFR pathway signaling	55	16	2.4 × 10^–5^	0.0012
Development_Estrogen-independent activation of ESR1 and ESR2	44	14	2.6 × 10^–5^	0.0013
Cell cycle progression in Prostate Cancer	39	13	2.8 × 10^–5^	0.0014
Development_WNT/Beta-catenin signaling in the nucleus	62	17	3.2 × 10^–5^	0.0015
Immune response_Fc epsilon RI pathway: Lyn-mediated cytokine production	87	21	3.3 × 10^–5^	0.0015
Development_Membrane-bound ESR1: interaction with growth factors signaling	45	14	3.4 × 10^–5^	0.0015
Altered Ca2 + handling in heart failure	35	12	4.2 × 10^–5^	0.0018
Signal transduction_Additional pathways of NF-kB activation (in the cytoplasm)	52	15	4.9 × 10^–5^	0.0019
Development_Endothelin-1/EDNRA signaling	52	15	4.9 × 10^–5^	0.0019
Signal transduction_Activation of PKC via G-Protein coupled receptor	52	15	4.9 × 10^–5^	0.0019
Cytoskeleton remodeling_Role of PKA in cytoskeleton reorganization	41	13	5.2 × 10^–5^	0.0020
NF-AT signaling in cardiac hypertrophy	65	17	6.2 × 10^–5^	0.0023
Apoptosis and survival_BAD phosphorylation	42	13	6.9 × 10^–5^	0.0025
Signal transduction_Calcium-mediated signaling	72	18	7.3 × 10^–5^	0.0025
Muscle contraction_Relaxin signaling pathway	48	14	7.6 × 10^–5^	0.0026
Cytoskeleton remodeling_Reverse signaling by Ephrin-B	32	11	8.5 × 10^–5^	0.0029
Neurophysiological process_Thyroliberin signaling	73	18	8.8 × 10^–5^	0.0029
POMC, alpha-MSH and AGRP in regulation of food intake and energy expenditure in obesity in hypothalamus	43	13	9.0 × 10^–5^	0.0029
Role of neuropeptides in pathogenesis of SCLC	67	17	9.4 × 10^–5^	0.0029
Transcription_CREB signaling pathway	49	14	9.7 × 10^–5^	0.0030
Signal transduction_Cyclic AMP signaling	38	12	1.0 × 10^–4^	0.0031
Immune response_IL-6 signaling pathway via MEK/ERK and PI3K/AKT cascades	74	18	1.1 × 10^–4^	0.0031
Cytoskeleton remodeling_CDC42 in cellular processes	23	9	1.2 × 10^–4^	0.0034
Neurophysiological process_Corticoliberin signaling via CRHR1	50	14	1.2 × 10^–4^	0.0034
Apoptotic pathways and resistance to apoptosis in lung cancer cells	56	15	1.2 × 10^–4^	0.0034
Tinnitus-associated changes in auditory pathway	82	19	1.4 × 10^–4^	0.0038
Cell adhesion_Histamine H1 receptor signaling in the interruption of cell barrier integrity	45	13	1.5 × 10^–4^	0.0041
Ca(2 +)-dependent NF-AT signaling in cardiac hypertrophy	57	15	1.5 × 10^–4^	0.0041
Signal transduction_Erk Interactions: Inhibition of Erk	34	11	1.6 × 10^–4^	0.0041
Development_Positive regulation of WNT/Beta-catenin signaling at the receptor level	64	16	1.8 × 10^–4^	0.0046

FDR, false discovery rate

## Discussion

In this study of 396 mother–newborn pairs from a predominantly urban, low-income, and multi-ethnic birth cohort in the USA, we observed significant correlations in genome-wide DNAm patterns, assessed by the latest Illumina Infinium MethylationEPIC BeadChip, between mothers (venous whole blood) and their newborns (cord whole blood); such correlations significantly differed by newborn sex, with mother–female pairs having relatively stronger correlations than mother–male pairs. These findings were replicated in both pre-pregnancy and early-pregnancy mother–newborn pairs from the IOWBC. In genome-wide analysis, we observed 89,267 DNAm sites with significant associations between maternal and neonatal methylation levels; 15 DNAm sites (11 of them on the X chromosome) showed significant interaction effect with newborn sex and were mapped to genes that have been associated with sex-specific disease/traits or early development. In addition, we also observed significant associations between differences in maternal-neonatal methylation levels (ΔDNAm) and newborn sex for 18,769 DNAm sites, most of which were on the X chromosome; these DNAm sites were mapped to 3,510 genes that were enriched to biological processes for neurodegenerative and psychological diseases, development, neurophysiological process, immune response, and sex-specific cancers.

In the context of the existing literature, this present study has made several new contributions to the field. As a major epigenetic regulation mechanism, neonatal DNAm changes have been associated with a large number of prenatal exposures, including environmental pollutants (e.g., air pollution, heavy metals, and chemicals [9–12]), maternal behavioral factors (e.g., smoking, alcohol, and substance use [13–16]), and maternal mental health conditions [18]. Additionally, DNAm changes have also been related to increased risk of many chronic diseases such as cancers [67], cardiovascular diseases [68, 69], and allergic diseases [70]. Moreover, DNAm changes caused by exogenous exposures can escape the major waves of epigenetic reprogramming during fertilization and gametogenesis and can be inherited intergenerationally [6]. However, to our knowledge, the intergenerational link in DNAm patterns has not been systematically investigated at the genome-wide level. In this study, we observed significant correlations for overall DNAm patterns between mothers and their newborns in an urban, multi-ethnic birth cohort in the USA; the observed correlations in matched mother–newborn pairs were significantly higher than that in random pairs, suggesting that family relatedness (genetics and/or epigenetics) drives intergeneration transmission of DNAm variation. In addition, DNAm sites showing significant mother–newborn associations had relatively larger *h*^*2*^, indicating that genetics may play an important role in the intergenerational inheritance of DNAm variation. Moreover, we also observed significant associations in mother–newborn methylation levels at a large number of DNAm sites, and > 50% of the mapped genes also showed significant correlations in mother–newborn expression. Therefore, our findings provided the first-hand evidence of the similarity of genome-wide DNAm patterns between generations in humans, as well as the potential impact on intergenerational correlation in gene expression.

DNAm sites are methylated differently by sex across the whole genome [25, 27–29], which could contribute to sex-biased gene expression and sexually differentiated traits [30, 31]. Substantial sex differences in DNAm levels across the whole genome have been demonstrated, especially on the X chromosome, in neonatal cord blood samples [56]. However, it is unknown whether the intergenerational inheritance of DNAm patterns differs by newborn sex. In this study, we observed significant sex differences in the mother–newborn correlations in overall DNAm patterns, especially the DNAm pattern on the X chromosome, with female newborns showing higher correlations with their mothers than male newborns. X-chromosomal DNAm sites also showed larger and more significant interaction effects with newborn sex. A potential explanation of these findings is female X-inactivation, a phenomenon where one of the X chromosomes is inactivated during early embryonic development in females [4], and the DNAm sites on the inactive X chromosome are predominantly methylated, while the DNAm sites on autosomes and the active X chromosome are predominantly unmethylated [71, 72]. This could also explain our finding that differences in mother–female methylation levels for X-chromosomal DNAm sites were close to 0, but male newborns had generally lower methylation levels than their mothers. In contrast, differences in mother–newborn methylation levels for autosomal DNAm sites were very similar between mother–female and mother–male pairs. Interestingly, many DNAm sites showed large differences in methylation levels between mothers and their newborns; such differences could be caused by age-related changes in DNAm status [25, 73]. However, potential factors contributing to the intergenerational differences in methylation levels deserve further investigation.

DNA methylation is an epigenetic regulatory mechanism that is involved in embryonic development and sexual differentiation [74, 75]. In mouse models, demethylation upstream of the Y-encoded sex-determining gene *Sry* during embryonic development leads to its expression, which breaks the male–female balance and directs sexual differentiation of bipotential cells toward male commitment [74]. In addition, the secreting of sex hormones (estrogen and testosterone) during early embryonic development drives sexual differentiation [76]. Sex hormones and their receptors can influence the expression of epigenetic modifiers and also interact with epigenetic modifiers, such as DNA methylation enzymes, impacting the epigenetic regulation of subsequent gene expression and leading to sexually differentiated traits [77]. Furthermore, animal experiments have demonstrated that manipulating DNA methylation process could impact sexual differentiation in the brain and further impact the behaviors in rodents [78]. In this study, we used pathway enrichment analysis to further examine the potential functions of genes whose DNAm sites showed significant associations between ΔDNAm and newborn sex. The top enriched pathways are involved in neurodegenerative and psychological diseases, development, neurophysiological process, immune response, and sex-specific cancers. Therefore, one might speculate that sex differences in the intergenerational inheritance of DNAm patterns could contribute to the development of sex differences in epigenetic regulations from the beginning of sexual differentiation. However, more studies are needed to systematically investigate this hypothesis.

Our study findings from two independent birth cohorts indicate the need for additional studies to line these findings with developmental origins and sex differences in pediatric and adult diseases. Alterations in DNA methylation status, also known as epimutations, have been related to the disease risk. A few human studies have indicated that epimutations could be inherited intergenerationally. A study from Saudi Arabia observed that epimutations on *BRCA1* and *MGMT* genes (both genes have been related to breast cancer) can be transmitted from mothers to female newborns [22], which could potentially transmit breast cancer risk from mothers to their daughters. Another study from France reported that epimutations of DNA mismatch repair genes, such as *MLH1* and *MSH2*, can be transmitted intergenerationally; epigenetic silencing of these genes has been related to increased risk of early-onset colorectal and endometrial cancers, and children who inherited these epimutations also developed early colonic tumors [23]. In addition, recent studies indicated that sex differences in DNA methylation status may contribute to sex differences in neurodegenerative diseases (e.g., Alzheimer’s disease) [79] and psychological disorders (e.g., depression and post-traumatic stress disorder) [80]. In this study, we performed genome-wide analysis to examine mother–newborn correlations in methylation levels. Our findings indicated 89,267 DNAm sites with significant mother–newborn correlations in methylation levels, and more than half of their mapped genes also showed significant mother–newborn correlations in expression levels. However, due to the lack of gene expression data, we cannot examine whether DNAm sites showed significant sex differences in mother–newborn correlations or differences also contribute to sex differences in mother–newborn correlations in gene expression level. Future studies are needed to reveal such a mechanism, as well as its contribution to the intergenerational inheritance of sex-biased disease risk.

This study has many strengths. First, we used data from a well-established urban birth cohort with a diverse race/ethnicity background in the USA and then replicated it in an independent birth cohort. Second, whole genome DNAm profiles of maternal and neonatal whole blood samples were performed using the latest Illumina Infinium MethylationEPIC BeadChip with over 850,000 DNAm sites. This state-of-the-art DNAm array provides comprehensive coverage of different functional areas across all autosomes and the X chromosome, including DNAm sites inside and outside of CpG islands, gene regulatory regions, non-CpG methylated sites identified in human stem cells, and differentially methylated sites identified in tumor versus normal, and other regulatory regions. Third, maternal characteristics and neonatal birth outcomes were obtained using standardized questionnaires or electronic medical records, which allowed adjustment and stratification of these important covariates in this study.

Our study also has limitations. First, the lack of paternal DNA samples makes us unable to estimate the intergenerational correlations for paternal–neonatal DNA methylation, and we also cannot evaluate whether paternal DNA methylation status contributes to the observed sex differences in mother–newborn correlations in DNA methylation. Therefore, future studies with paternal DNA methylation are needed to evaluate sex differences in father–newborn correlations in DNA methylation status. Second, maternal DNA samples were collected 24–48 h postpartum. It is possible that maternal DNA methylation status was affected by the processes of pregnancy and delivery, as previous studies have reported that pregnancy could reprogram maternal DNA methylation status and persist after childbirth [81]. However, our main findings were replicated in the IOWBC, where the maternal blood samples were collected at 18 years old (pre-pregnancy pairs) and 10–21 weeks of gestation (early-pregnancy pairs), indicating that the observed sex differences in mother–newborn correlations/differences in DNAm were not affected by the timing of maternal blood sample collection and the processes of pregnancy and delivery. Additionally, fetuses inherit parental DNA methylation markers directly from germline DNA. Therefore, although we adjusted for several covariates (i.e., maternal age at delivery, maternal race/ethnicity, maternal smoking, preterm birth, type of delivery, and SVs), it is unclear whether other pregnancy-related factors, such as maternal pre-pregnancy body mass index and weight gain, pregnancy complications, and nutritional factors, impact the observed mother–newborn correlations/differences in DNA methylation status. Further studies are needed to examine the impact of these factors on intergenerational correlation in DNAm. Third, this study was conducted in a US urban birth cohort that recruited predominantly low-income, Black pregnant women, which may limit generalizable but is also a strength in investigating an understudied population. Similar results were observed in the IOWBC, which included only White pregnant women from the UK. But such replication was limited by the number of DNAm sites available for analyses. More studies with DNAm data profiled using the MethylationEPIC BeadChip are needed to replicate our findings. Finally, our study lacks a replication sample. For a previously reported gene (*BRCA1*) whose methylation status was shown to be transmitted from mothers to daughters, we observed 6 methylation sites that demonstrated significant mother–newborn correlations (Additional file 2: Table S1); this supports that our findings are potentially robust. However, more studies are needed to replicate our findings, and replication studies in additional populations are needed.

## Conclusions

Using data from 2 independent birth cohorts, the BBC and IOWBC, our study demonstrated sex differences in the intergenerational link in DNA methylation status across the whole genome between mothers and their newborns. First, we observed significant mother–newborn correlations in genome-wide DNAm pattern, and such correlations significantly differed by newborn sex, with mother–female pairs having relatively stronger correlations than mother–male pairs. Second, we observed significant associations between maternal and neonatal methylation levels at 89,267 DNAm sites on autosomes and the X chromosome; in sex interaction analysis, we observed 4 autosomal and 11 X-chromosomal DNAm sites showed significant interaction effects with newborn sex, and these DNAm sites were mapped to genes that have been associated with sex-specific disease/traits or early development. Third, we calculated differences in maternal-neonatal methylation levels and estimated their associations with newborn sex, and 18,769 DNAm sites (14,482 of them were on the X chromosome) showed significant associations, and these DNAm sites were mapped to 3,510 genes enriched to biological processes for neurodegenerative and psychological diseases, development, neurophysiological process, immune response, and sex-specific cancers. Our findings indicate that the intergenerational correlations in DNAm status between mothers and their newborns were strong and significantly differ by sex, which could offer new insight into developmental origins of many pediatric and adult diseases with remarkable sex differences in risk, such as autism, attention-deficit/hyperactivity disorder, cardiovascular diseases, and cancer. Future studies are required to estimate the contribution of paternal methylation levels on the intergenerational link in DNAm status. More studies are needed to investigate whether the observed sex differences in mother–newborn correlations in DNAm contribute to the development of sex differences in disease risk.

## Methods

### Study population

#### Boston birth cohort (BBC)

The study included 396 mother–newborn pairs from the BBC, an ongoing birth cohort study initiated in 1998 at the Boston Medical Center, Boston, MA [82]. Mothers who delivered live singletons were eligible and were invited to participate within 1 to 3 days after delivery. Mothers who provided written informed consent were enrolled and interviewed using a standardized questionnaire. Pregnancies that were a result of in vitro fertilization, multiple gestations (e.g., twins or triplets), fetal chromosomal abnormalities or major birth defects, and preterm deliveries due to non-obstetric factors (e.g., trauma) were excluded. In addition, participants of the BBC are predominantly low-income patients with oversampling of preterm delivery and low birthweight.

#### The Isle of Wight birth cohort (IOWBC)

The IOWBC was initiated at the David Hide Asthma and Allergy Research Centre on the Isle of Wight, UK, and 1,456 of all children (n = 1,536) born between January 1989 and February 1990 were followed up at 1, 2, 4, 10, 18 and 26 years of age (F1-generation) [83]. Between 2010 and 2015, 431 offspring of the F1-generation were recruited (F2-generation) [83, 84]. In this study, we mother–newborn pairs were formed based on data from the F1-generation (i.e., mothers) and the F2-generation (i.e., newborns). For mothers who delivered multiple times during the study period, only the first live birth of each mother was included.

### DNA methylation profiling

In the BBC, maternal blood samples were obtained within 24–72 h after delivery. Umbilical cord blood samples were collected at delivery. Blood samples were stored in a − 80 °C freezer until analysis. DNA methylation profiling on maternal and neonatal blood samples was conducted using the Illumina Infinium MethylationEPIC BeadChip (Illumina, San Diego, CA) at the University of Minnesota Genomics Center, and methylation levels of 865,859 methylation sites were measured. Maternal blood (n = 418), umbilical cord blood (n = 963) and 29 duplicates (8 maternal samples and 21 cord blood samples) samples were randomly placed in 96-well DNA plates. Laboratory personnel were blinded to the sample placement. Details on DNAm profiling and quality control (QC) have been described previously [14]. Briefly, the systematic QC was performed using standard analytic pipelines with the R package ‘minfi’ [85], and data QC steps were performed in maternal blood and cord blood samples, along with mother–child dyad and newborn sex information. Multi-dimensional scaling and intensity plots were used to evaluate outlier samples, and multi-dimension scaling plots were used to confirm maternal blood samples, male cord blood samples, and female cord blood samples. Samples whose DNAm-derived sex was inconsistent with the documented sex in medical records were excluded from the subsequent analyses. We also removed samples with missing data in > 2% DNAm sites. In addition, DNAm sites were removed if having 1) a detection p > 0.01 in over 5% of the samples or 2) an annotated SNP at the measured or extension site, or that overlapped with the probe, and/or that potentially cross-hybridized to other genomic locations. After quality control steps, 721,331 DNAm sites, with 704,552 on autosomes and 16,779 on the X chromosome, for 396 mother–newborn pairs were eligible for subsequent analyses.

In the IOWBC, maternal DNA (the F1-generation) was extracted from peripheral blood samples collected at age 18 years (n = 249) or during early pregnancy (10–21 weeks of gestation; n = 132), and the neonatal DNA (the F2-generation) was extracted from cord blood samples (n = 192). The Illumina Infinium HumanMethylation450 BeadChip (Illumina, San Diego, CA) was used for the DNAm profiling of 272 maternal samples at age 18 years, 130 maternal samples collected at early pregnancy, and 129 cord blood samples, while the MethylationEPIC BeadChip (Illumina, San Diego, CA) was used for 27 maternal samples at age 18 years, 2 maternal samples at early pregnancy, and 63 cord blood samples. DNA samples were randomly distributed on the arrays to eliminate batch effects. Arrays were processed using a standard protocol with multiple identical control samples assigned to each bisulfite conversion batch to assess assay variability. Probes not reaching a detection p-value of 1 × 10^–16^ in at least 95% of samples were excluded. DNAm value was calculated by the ratio of methylated over the sum of methylated and unmethylated probes [86]. Quantile normalization was applied to DNAm intensities as suggested in the CPACOR pipeline [87]. Data pre-processing was performed on shared DNAm sites between the 2 BeadChips. As a result, this study included 48 pre-pregnancy mother–newborn pairs (i.e., matched maternal blood samples at age of 18 years [F1] and cord blood samples [F2]) with 218,259 DNAm sites (including 208,573 autosomal and 9,686 X-chromosomal DNAm sites) available for data analysis, and 93 early-pregnancy mother–newborn pairs (i.e., matched maternal blood samples in early pregnancy [F1] and cord blood samples [F2]) with 401,539 DNAm sites (including 391,902 autosomal and 9,637 X-chromosomal DNAm sites).

### Definition of maternal and neonatal characteristics

Information on maternal socio-demographic characteristics, psychological stress, lifestyle and prenatal multivitamin intake were assessed through standard maternal questionnaire interview for both BBC and IOWBC [82–84]. In the BBC, we surveyed maternal smoking status by asking whether a pregnant woman used tobacco 1) in the 6 months before finding out being pregnant, 2) in the first 3 months of pregnancy, 3) in the middle 3 months of pregnancy, or 4) in the last 3 months of pregnancy. Mothers who did not use tobacco in any of these time windows were defined as “non-smokers,” and mothers who used tobacco in at least one of these time windows were defined as “smokers.” Maternal race/ethnicity was self-reported. “Black” indicates self-identified African American and Haitian, and “non-Black” indicates Hispanic, White, and others. In the IOWBC, all women included were White.

Clinical data such as gestational age, pregnancy complications, type of delivery, birth order, and birth weight were obtained through electronic medical records (BBC) or hospital notes (IOWBC). In the BBC, gestational age was assessed by early prenatal ultrasound (< 20 weeks) or based on the first day of the last menstrual period as recorded in maternal electronic medical records if early prenatal ultrasound was not available [82]. Preterm birth was defined as birth occurring at < 37 weeks of gestation.

### Statistical analysis

We used a flowchart to illustrate the steps of our statistical analyses in the BBC (Additional file 1: Figure S9).

#### Sample-wise correlations between maternal and neonatal DNAm patterns

To investigate the overall genome-wide similarity of DNAm pattern between mother and newborn pairs, we first calculated Spearman’s rank correlation coefficients for each mother–newborn pair. Additionally, to characterize the background distribution of intergenerational correlation without family relatedness (under the NULL hypothesis of no family relatedness) while accounting for correlation structure among DNAm sites, we randomly shuffled cord blood samples with unrelated maternal blood samples, matching by newborn sex, and calculated the Spearman’s rank correlation coefficients for each unrelated mother–newborn pair; this process was repeated for 10 times (i.e., a given newborn was randomly paired to an unrelated mother in an iteration), and the average correlation coefficient of the 10 iterations was considered as the background intergenerational correlation coefficient without family relatedness. Maternal-neonatal correlations were also computed separately by autosomal and X-chromosomal DNA sites. Then, the Student’s paired *t* test was used to estimate whether the observed distribution of mother–newborn correlation coefficients was different from the background correlation coefficients. Finally, we calculated the mother–newborn correlation coefficients stratified by newborn sex to examine sex differences in correlations between maternal and neonatal DNAm patterns, and Student’s two-sample *t* test was used to compare correlation coefficients between sexes. As a replication, the same analyses were performed in the IOWBC pre-pregnancy and early-pregnancy mother–newborn pairs.

#### Site-wise correlations between maternal and neonatal methylation levels

After examining the sample-wise similarity, we investigated the mother–newborn correlations at individual DNAm sites. For each DNAm site, we first computed the Spearman’s rank correlation coefficient between maternal and neonatal methylation values.

We first estimated surrogate variables (SV) to capture potential confounding effects of batches, cell compositions, population stratification, and other unknown confounders using R package SmartSVA [88]. In this analysis, we considered neonatal DNAm (DNAm_neonatal_) as the outcome and maternal DNAm (DNAm_maternal_) as the exposure. We thus calculated the SVs using the DNAm_neonatal_ as the outcome in the SmartSVA model, adjusting for maternal age at delivery, maternal race/ethnicity, maternal smoking, preterm birth, and type of delivery. After calculation, 36 SVs were generated. We included the type of delivery as a covariate to account for the timing of sampling, because, in the BBC, blood samples for women who went through natural delivery were collected within 24–48 h after delivery, and blood samples for women who went through C-section were collected after 48 h but typically < 72 h after delivery. Methylation values of each DNAm site for mothers and their newborns were standardized separately using inverse normal transformation.

To account for potential confounders and examine association heterogeneity by newborn sex, we applied linear regression models to estimate the association between neonatal (as the outcome) and maternal (as the independent variable) methylation levels of each DNAm site adjusting for newborn sex and covariates (maternal age at delivery, maternal race/ethnicity, maternal smoking, preterm birth, type of delivery, and all 36 SVs). We performed a full model including covariate-maternal methylation interaction terms (DNAm_neonatal_ ~ DNAm_maternal_ + newborn sex + DNAm_maternal_ × newborn sex + ∑covariate + ∑DNAm_maternal_ × covariate + ∑SV) and a reduced model (DNAm_neonatal_ ~ newborn sex + ∑covariate + ∑SV) for each DNAm site, and then conducted Likelihood Ratio Test to assess the mother–newborns associations in methylation levels at each DNAm site. We calculated false discovery rate (FDR) to assess statistical significance accounting for 721,331 DNAm sites. A DNAm site with an FDR < 0.05 was considered significant and was mapped to the corresponding genes based on its genomic position. To examine the potential contribution of genetics on the observed mother–newborn associations, we obtained the *h*^*2*^ of DNAm levels from a published study on DNAm quantitative trait loci (mQTL) [25], where 341,069 autosomal DNAm sites included in our study were available. Finally, we estimated the associations in the expression levels of their corresponding genes between maternal and neonatal blood samples using data from a publicly available repository (GSE27272; the expression of 24,526 genes in maternal blood and cord blood samples from 60 mother–newborn pairs was measured using the Illumina HumanRef-8v3.0 BeadChip. Maternal and neonatal samples were collected in the Ceske Budejovice Hospital, Czech Republic, between November 2008 and March 2009) [32].

To examine sex differences in maternal-neonatal associations, we performed hypothesis test for the DNAm_maternal_ × newborn sex interaction term in the full model; DNAm sites with an FDR < 0.05 were considered significant sex interaction and were mapped to their corresponding genes based on their genomic positions. As a replication, the same analyses were performed for DNAm sites with an FDR < 0.05 in sex interaction analysis in the IOWBC pre-pregnancy and early-pregnancy mother–newborn pairs.

#### Differences in maternal and neonatal methylation levels and associations with newborn sex

As the cord blood samples represent the DNAm status at the very beginning of life, differences in methylation levels between maternal whole blood and their offspring’s cord blood are also important to investigate. Differences between maternal and neonatal methylation values were calculated as ΔDNAm = DNAm_neonatal_ – DNAm_maternal_ for each DNAm site. Treating the ΔDNAm as the outcome, we re-estimated SVs to capture potential unobserved confounding effects using R package SmartSVA [88]. In this analysis, we considered ΔDNAm as the outcome and newborn sex as the exposure. We thus calculated the SVs using ΔDNAm as the outcome in the SmartSVA model, adjusting for maternal age at delivery, maternal race/ethnicity, maternal smoking, preterm birth, and type of delivery. After calculation, 37 SVs were generated. We then applied an inverse normal transformation to standardize the ΔDNAm for each DNAm site to avoid potential effects from outliers. Linear regression models were then used to estimate the association between ΔDNAm and newborn sex for each DNAm site, adjusting for maternal age at delivery, maternal race/ethnicity, maternal smoking, preterm birth, type of delivery, and all 37 SVs. DNAm sites with an FDR_sex_ < 0.05 were considered significant and were mapped to their corresponding genes based on their genomic positions. For DNAm sites with a FDR_sex_ < 0.05 and available in the IOWBC pre-pregnancy or early-pregnancy mother–newborn pairs, we performed the same analyses as a replication. Regression coefficients from the BBC and IOWBC were compared to examine the concordance.

To examine the potential contribution of genetics on DNAm sites showing significant associations between ΔDNAm and newborn sex, we obtained the *h*^*2*^ of DNAm levels from a published study on mQTL [25], where 341,069 autosomal DNAm sites included in our study were available. To further examine the potential functions of these identified corresponding genes, we performed pathway enrichment analyses to examine the potential functions for significant DNAm sites using MetaCore (https://portal.genego.com/), an online analytical tool for biological pathway analysis.

## Supplementary Information

**Additional file 1.** Supplementary Figures.

**Additional file 2.** Supplementary Tables.

## Data Availability

The data, data dictionary, and analytical programs for this manuscript are not currently available to the public. However, they can be made available upon reasonable request and after the review and approval of the institutional review board.

## Abbreviations

DNAm: DNA methylation
CpG: Cytosine–phosphate–guanine dinucleotide
BBC: Boston birth cohort
IOWBC: Isle of Wight birth cohort
QC: Quality control
SV: Surrogate variables
*h*^*2*^: Heritability
ΔDNAm: Differences between maternal and neonatal methylation levels for each DNAm site
TSS: Transcriptional start site
mQTL: DNAm quantitative trait loci
