# Supplementary material for: Sex differences in the intergenerational link between maternal and neonatal whole blood DNA methylation: a genome-wide analysis in 2 birth cohorts
Source: Clin Epigenetics. 2023 Mar 25;15:51. doi: 10.1186/s13148-023-01442-8 (PMC10040137; doi:10.1186/s13148-023-01442-8)

Supplementary materials

Sex Differences in the Intergenerational Link Between Maternal and Neonatal Whole Blood DNA Methylation: A Genome-wide Analysis in 2 Birth Cohorts

Jie Hu[1,2†], Xin Xu[2†], Jun Li[2,3], Yu Jiang[4], Xiumei Hong[5], Kathryn M. Rexrode[1], Guoying Wang[5], Frank B. Hu[2,3,6], Hongmei Zhang[4], Wilfried J. Karmaus[4], Xiaobin Wang[5,7], Liming Liang[2,8]

[†] These authors contributed equally.

Corresponding author

Liming Liang, PhD

655 Huntington Avenue, Building 2, Room 207, Boston, MA 02115, USA.

Email: lliang@hsph.harvard.edu

Office: +1 (617) 432-5896

Affiliations

[1] Division of Women's Health, Department of Medicine, Bigham and Women's Hospital and Harvard Medical School, Boston, MA.

[2] Department of Epidemiology, Harvard T.H. Chan School of Public Health, Boston, MA.

[3] Department of Nutrition, Harvard T.H. Chan School of Public Health, Boston, MA.

[4] Division of Epidemiology, Biostatistics, & Environmental Health, School of Public Health, University of Memphis, Memphis, TN.

[5] Center on the Early Life Origins of Disease, Department of Population, Family and Reproductive Health, Johns Hopkins University Bloomberg School of Public Health, Baltimore, MD.

[6] Channing Division of Network Medicine, Department of Medicine, Brigham and Women's Hospital and Harvard Medical School, Boston, MA.

[7] Department of Pediatrics, Johns Hopkins University School of Medicine, Baltimore, MD.

[8] Department of Biostatistics, Harvard T.H. Chan School of Public Health, Boston, MA.

Supplementary Figure S1. Distributions of overall maternal-newborn correlations in DNAm, stratified by newborn sex.

Overall mother-newborn correlations comparing paired maternal-newborn samples (marked as red) and randomly paired maternal-newborn samples (computed from 10 permutation; marked as grey), stratified by A-C) mother-male and B-D) mother-female pairs.

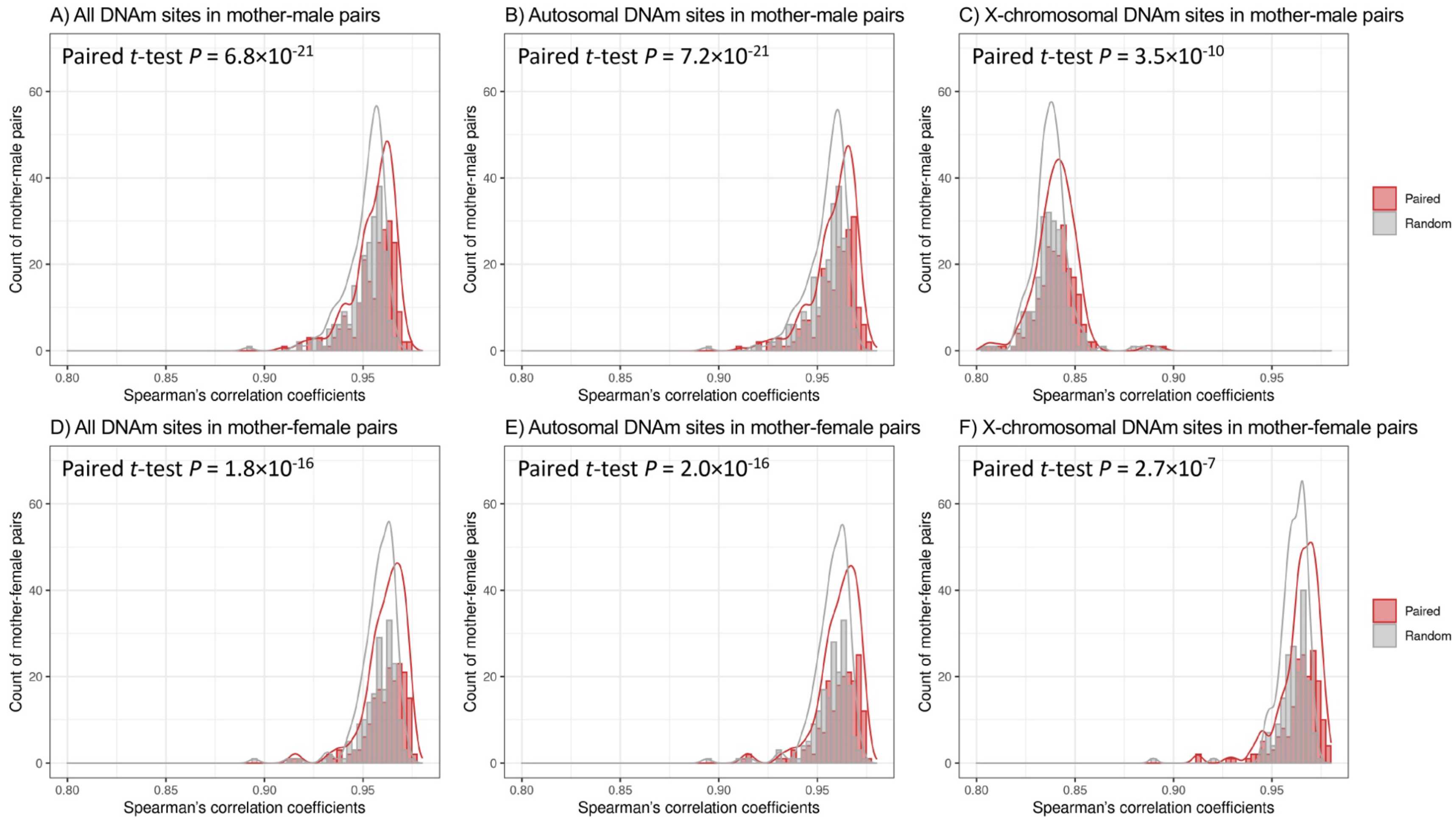

Supplementary Figure S2. Distributions of overall maternal-newborn correlations in DNAm by newborn sex in the BBC and IOWBC.

Overall mother-newborn correlations comparing between newborn sex for all, autosomal, and X-chromosomal DNAm sites in A-C) mother-newborn pairs in the BBC, D-F) pre-pregnancy mother-newborn pairs in the IOWBC, and G-I) early-pregnancy mother-newborn pairs in the IOWBC.

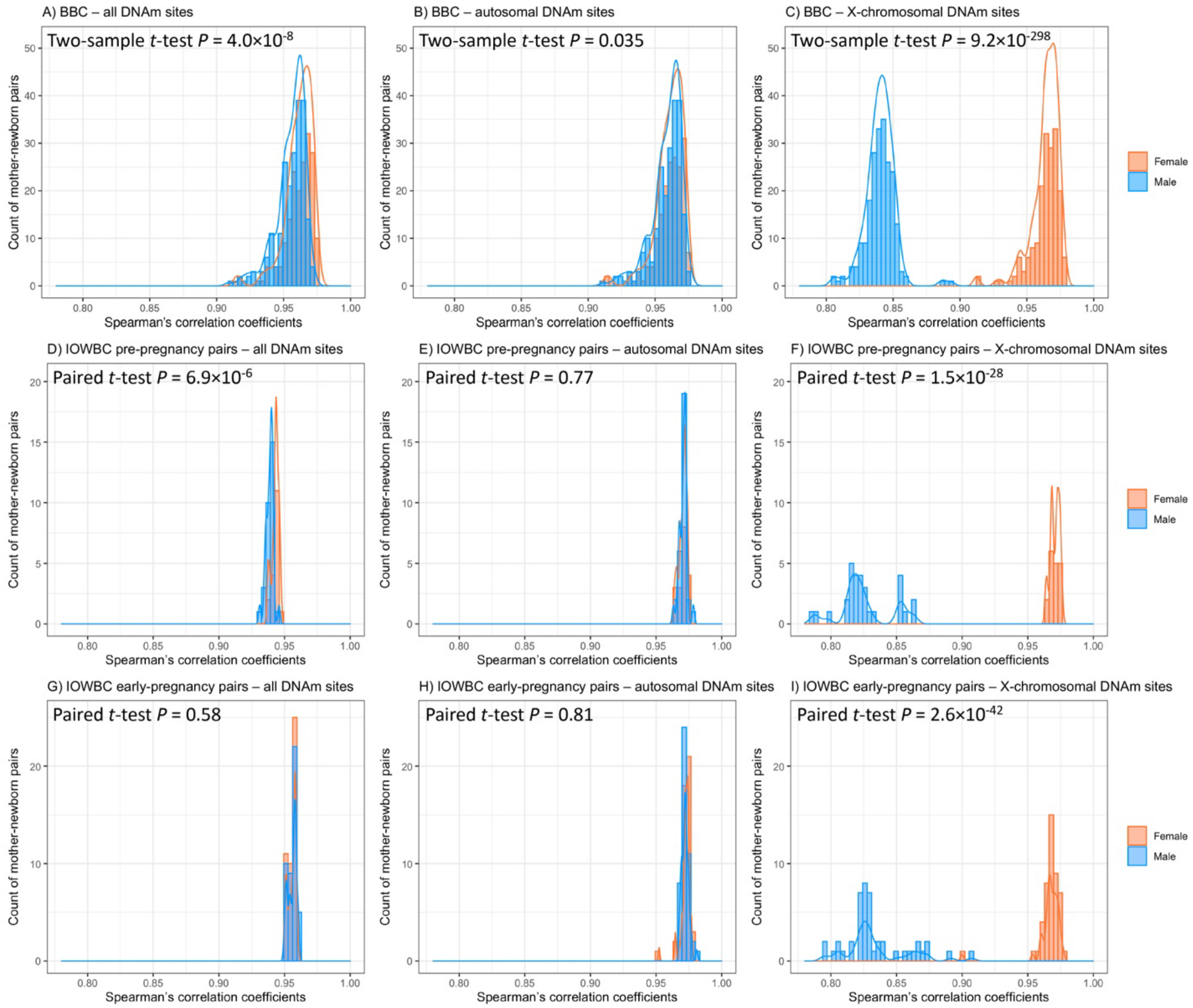

Supplementary Figure S3. Distribution of p-values from Likelihood Ratio Test that estimated mother-newborn associations in methylation levels at individual DNAm sites, by the h^2 of each DNAm site.

The X-axis is the heritability (h^2) of individual DNAm sites based on a published study on DNAm quantitative trait loci (van Dongen et al. 2016; PMID: 27051996), but only the heritability of autosomal DNAm sites was available. The Y-axis is the $-\log_{10}$(p-values) from Likelihood Ratio Test that estimated the associations in methylation levels at individual DNAm sites between mothers and their newborns. The red horizontal lines marks FDR=0.05.

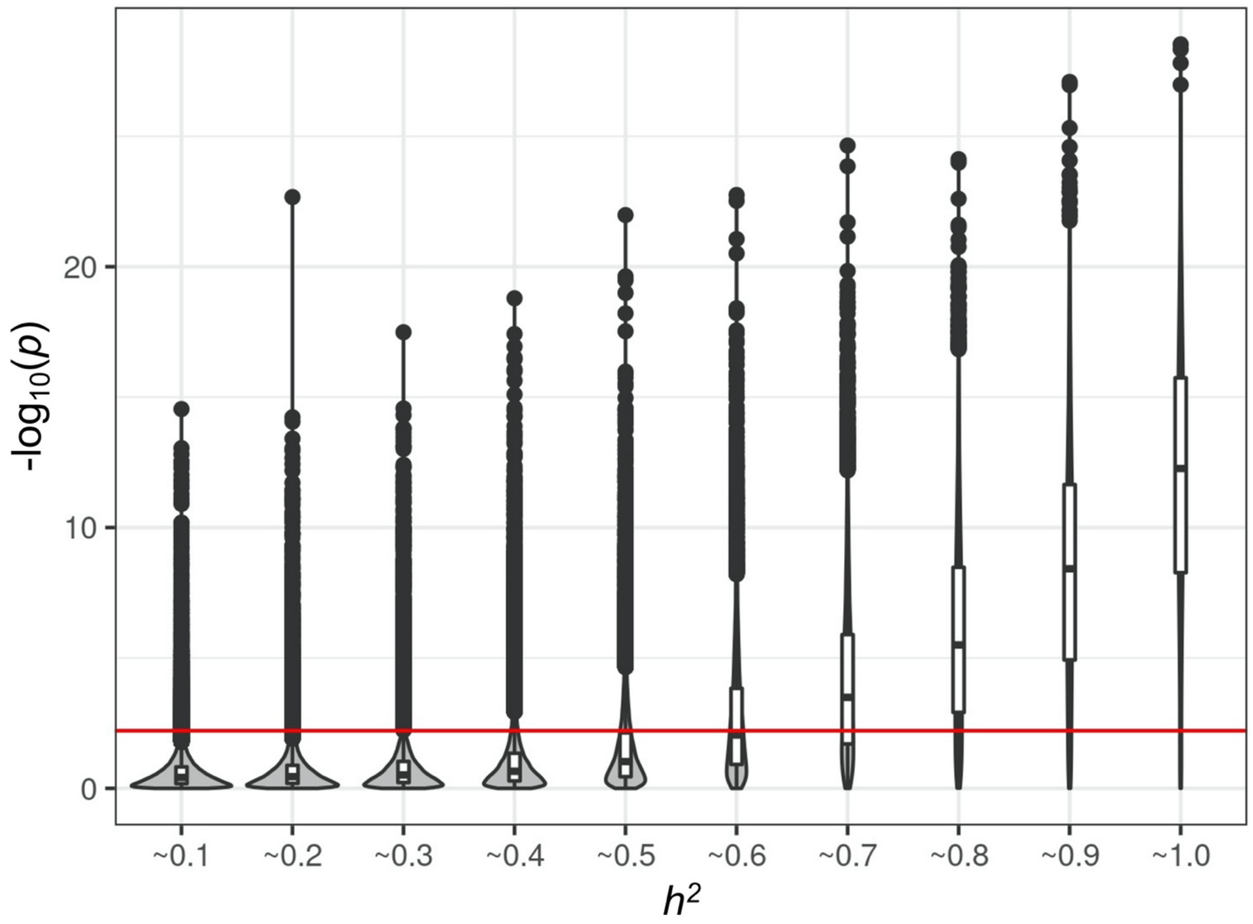

Supplementary Figure S4. Numbers of DNAm sites showing significant mother-newborn associations in methylation levels from Likelihood Ratio Test, by the h^2 of each DNAm site.

The X-axis is the heritability (h^2) of individual DNAm sites based on a published study on DNAm quantitative trait loci (van Dongen et al. 2016; PMID: 27051996), but only the heritability of autosomal DNAm sites was available. The Y-axis is the numbers of DNAm sites. Blue columns are DNAm sites showing nonsignificant mother-newborn associations (FDR≥0.05) in Likelihood Ratio Test, and grey columns are DNAm sites showing significant mother-newborn associations (FDR<0.05). The table below showed the numbers for DNAm showing significant or nonsignificant mother-newborn associations in each h^2 category.

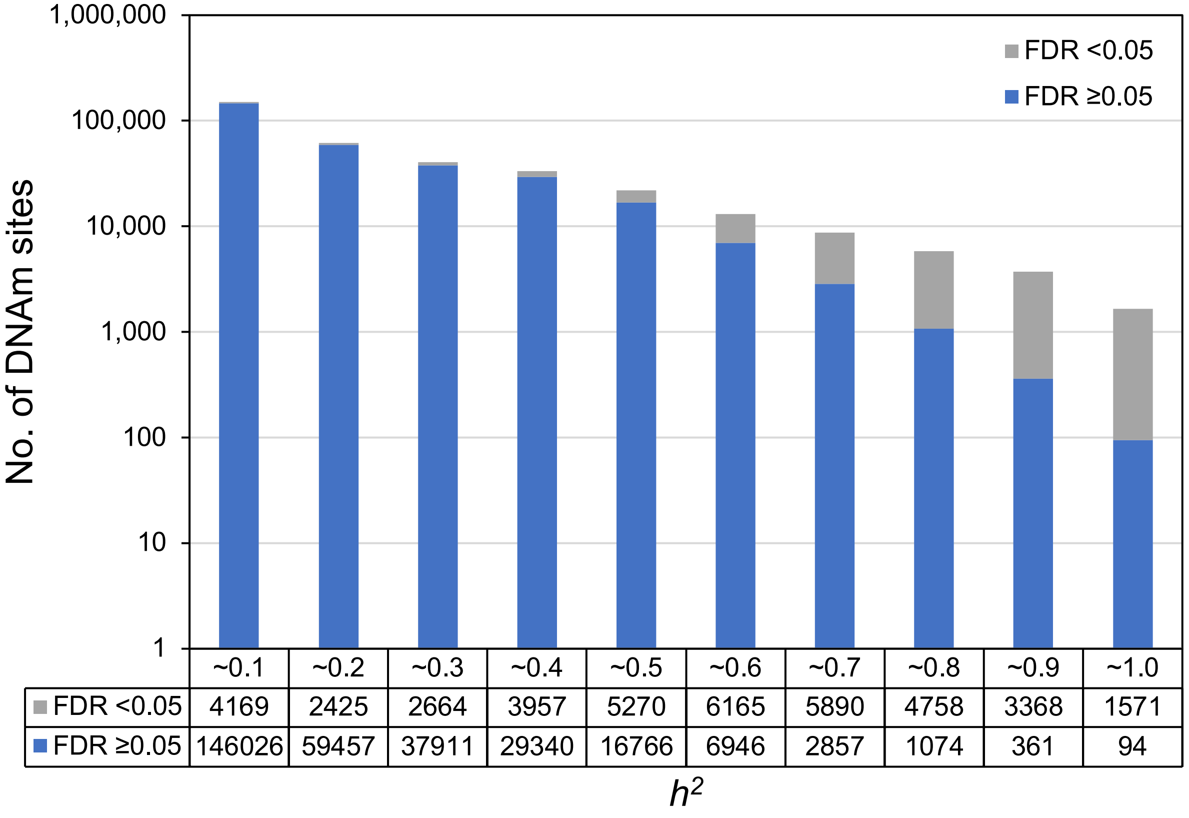

	~0.1	~0.2	~0.3	~0.4	~0.5	~0.6	~0.7	~0.8	~0.9	~1.0
FDR <0.05	4169	2425	2664	3957	5270	6165	5890	4758	3368	1571
FDR ≥0.05	146026	59457	37911	29340	16766	6946	2857	1074	361	94

Supplementary Figure S5. Concordance of results from the main and sensitivity analyses estimating mother-newborn associations in methylation levels for individual DNAm sites.

Distributions of A) p-values from the Likelihood Ratio Test, B) regression coefficients for the interaction term between maternal methylation levels and newborn sex, and C) p-values for the interaction term between maternal methylation levels and newborn sex from both main and sensitivity analyses were shown. The X-axis represented results for main analysis (i.e., models were adjusted for maternal age at delivery, maternal race/ethnicity, maternal smoking, preterm birth, type of delivery, and surrogate variables). The Y-axis represented results from the sensitivity analysis additionally adjusting for birth order.

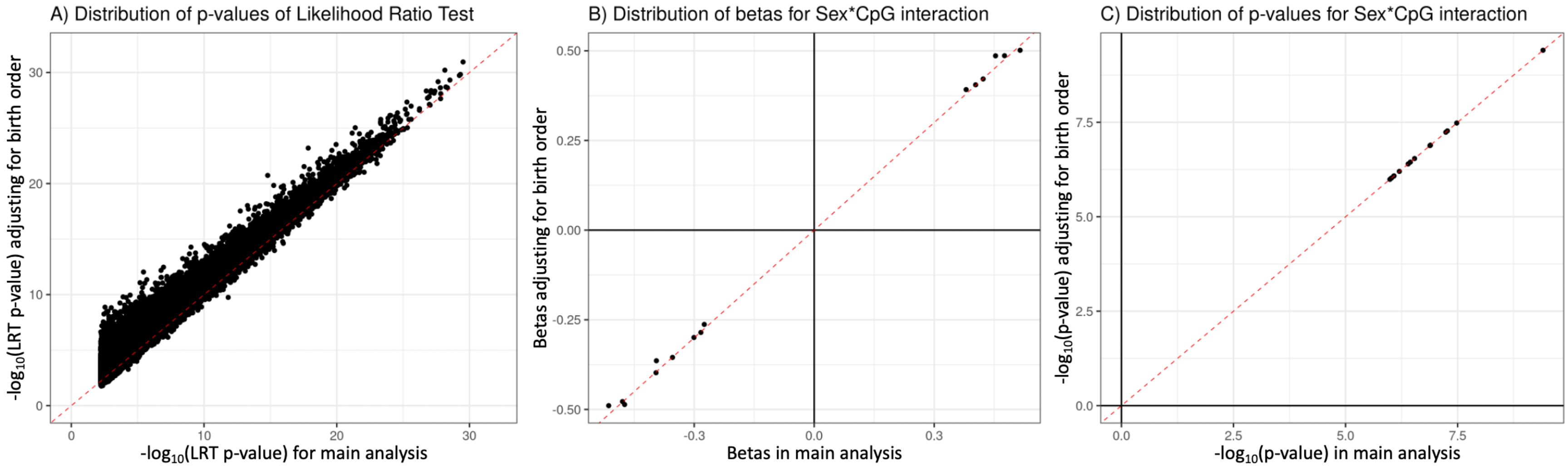

Supplementary Figure S6. Distribution of A) p-values and B) regression coefficients for associations between ΔDNAm and newborn sex at individual DNAm sites, by the h^2 of each DNAm site.

ΔDNAm represents differences in maternal-neonatal methylation levels of individual DNAm sites. The X-axis is the heritability (h^2) of individual DNAm sites based on a published study on DNAm quantitative trait loci (van Dongen et al. 2016; PMID: 27051996), but only the heritability of autosomal DNAm sites was available. The Y-axis is the A) $-\log_{10}$(p-values) and B) regression coefficients (betas) for associations between ΔDNAm and newborn sex. The red horizontal lines marks FDR=0.05.

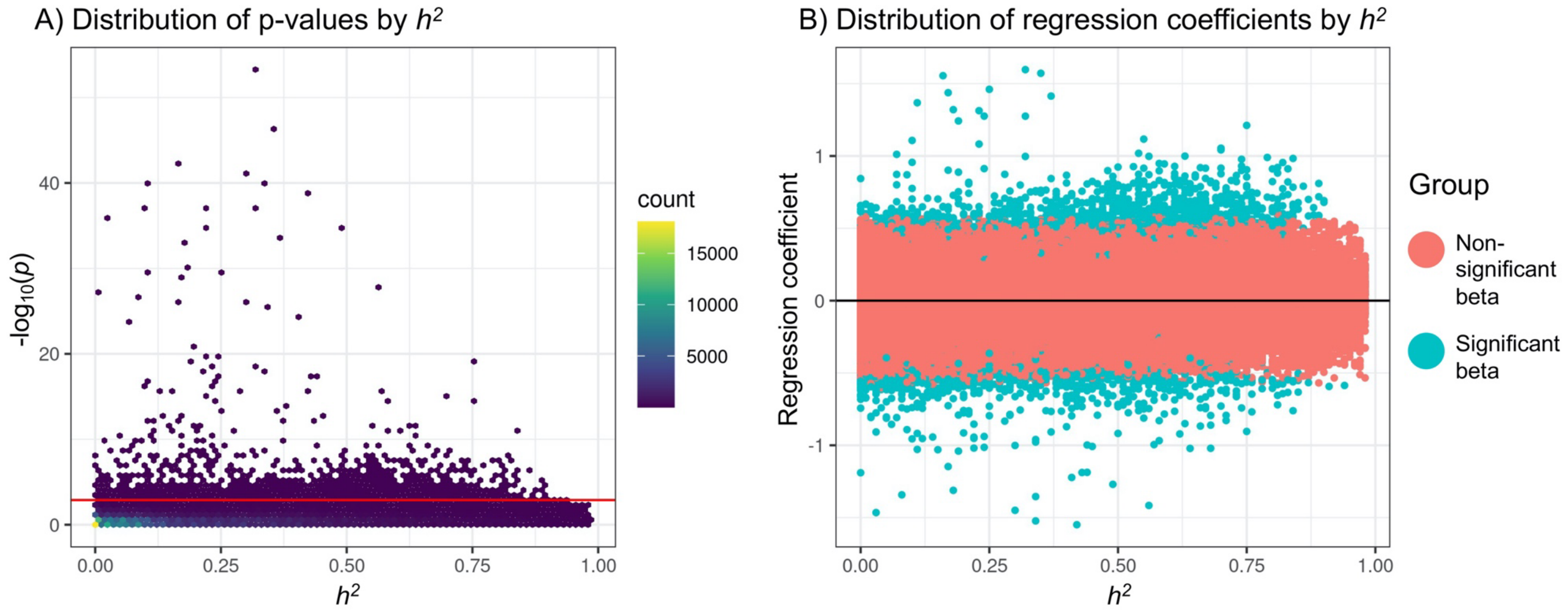

Supplementary Figure S7. Concordance of regression coefficients between BBC and IOWBC pre-pregnancy pairs for associations between ΔDNAm and newborn sex.

Regression coefficients for associations between ΔDNAm and newborn sex were compared between the BBC (primary analysis) and pre-pregnancy pairs from the IOWBC (replication) in A) all available 8,896 DNAm sites, and B) 1,245 DNAm sites showed a $p<0.05$ in the IOWBC. P-values for chi-square test for concordance were presented. C) DNAm sites with significant associations between ΔDNAm and newborn sex ($p<0.05$) in the IOWBC were enriched, with a $p<2.2\times10^{-16}$ in the Kolmogorov-Smirnov Test.

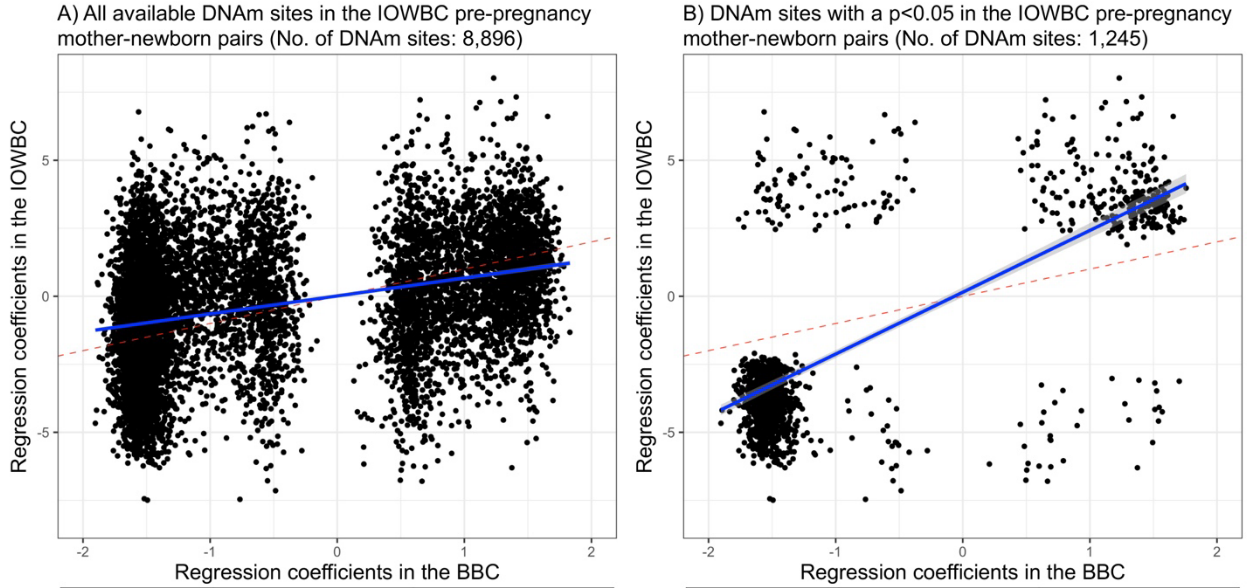

	$\beta_{BBC} > 0$	$\beta_{BBC} < 0$
$\beta_{IOWBC} > 0$	**1,960 (22.0%)**	2,069 (23.3%)
$\beta_{IOWBC} < 0$	1,180 (13.3%)	**3,687 (41.4%)**

Chi-square test for concordance $P = 5.2\times10^{-127}$

	$\beta_{BBC} > 0$	$\beta_{BBC} < 0$
$\beta_{IOWBC} > 0$	**253 (20.3%)**	98 (7.9%)
$\beta_{IOWBC} < 0$	33 (2.6%)	**861 (69.2%)**

Chi-square test for concordance $P = 6.7\times10^{-147}$

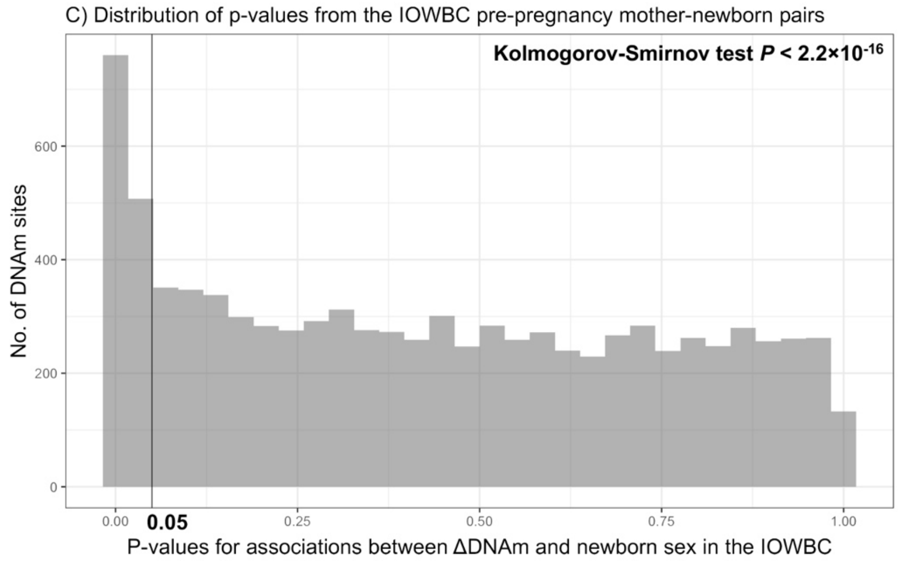

Supplementary Figure S8. Concordance of regression coefficients between BBC and IOWBC early-pregnancy pairs for associations between ΔDNAm and newborn sex.

Regression coefficients for associations between ΔDNAm and newborn sex were compared between the BBC (primary analysis) and early-pregnancy pairs from the IOWBC (replication) in A) all available 9,986 DNAm sites, and B) 1,827 DNAm sites showed a $p<0.05$ in the IOWBC. P-values for chi-square test for concordance were presented. C) DNAm sites with significant associations between ΔDNAm and newborn sex ($p<0.05$) in the IOWBC were enriched, with a $p<2.2\times10^{-16}$ in the Kolmogorov-Smirnov Test.

A) All available DNAm sites in the IOWBC early-pregnancy mother-newborn pairs (No. of DNAm sites: 9,986)

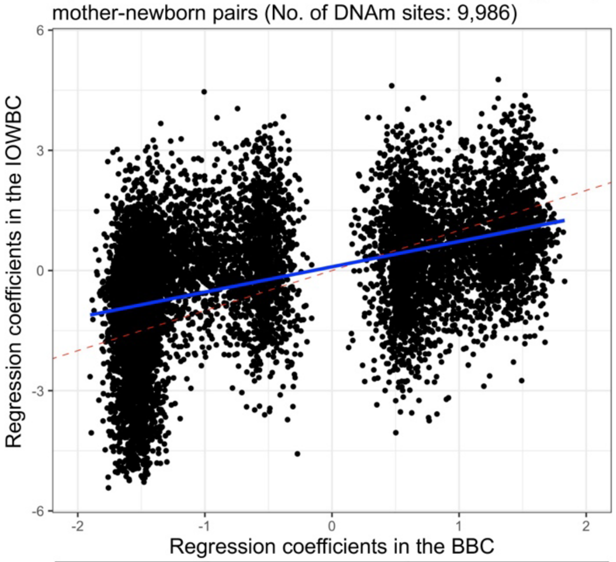

	$\beta_{BBC} > 0$	$\beta_{BBC} < 0$
$\beta_{IOWBC} > 0$	**2.611 (26.1%)**	2,253 (22.6%)
$\beta_{IOWBC} < 0$	1,155 (11.6%)	**3,967 (39.7%)**

Chi-square test for concordance *P* = 7.9×10^{-226}

B) DNAm sites with a p<0.05 in the IOWBC early-pregnancy mother-newborn pairs (No. of DNAm sites: 1,827)

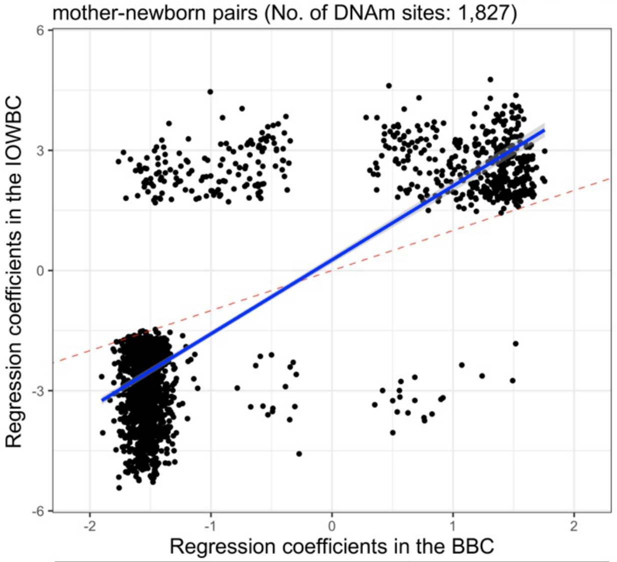

	$\beta_{BBC} > 0$	$\beta_{BBC} < 0$
$\beta_{IOWBC} > 0$	**414 (22.7%)**	143 (7.8%)
$\beta_{IOWBC} < 0$	20 (1.1%)	**1,250 (68.4%)**

Chi-square test for concordance *P* = 4.8×10^{-248}

C) Distribution of p-values from the IOWBC early-pregnancy mother-newborn pairs

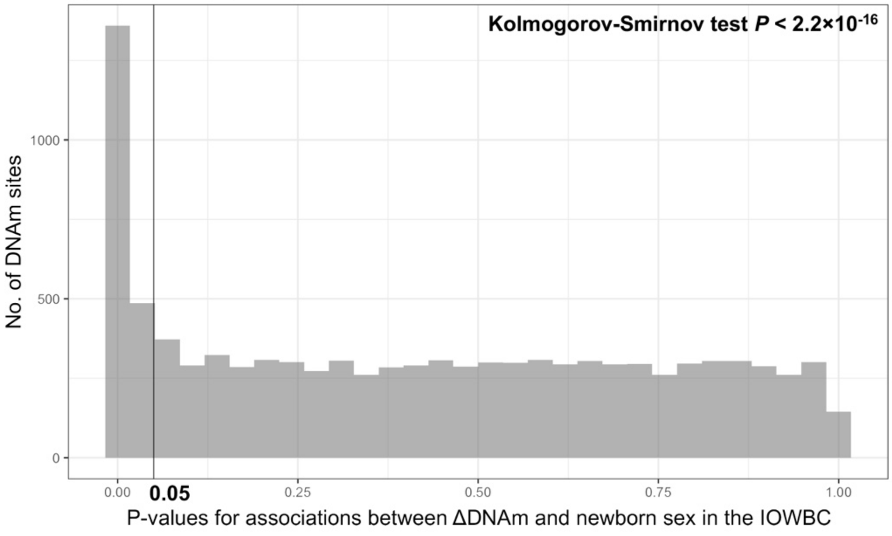

Supplementary Figure S9. Flow chart for analysis steps in the Boston Birth Cohort (BBC).

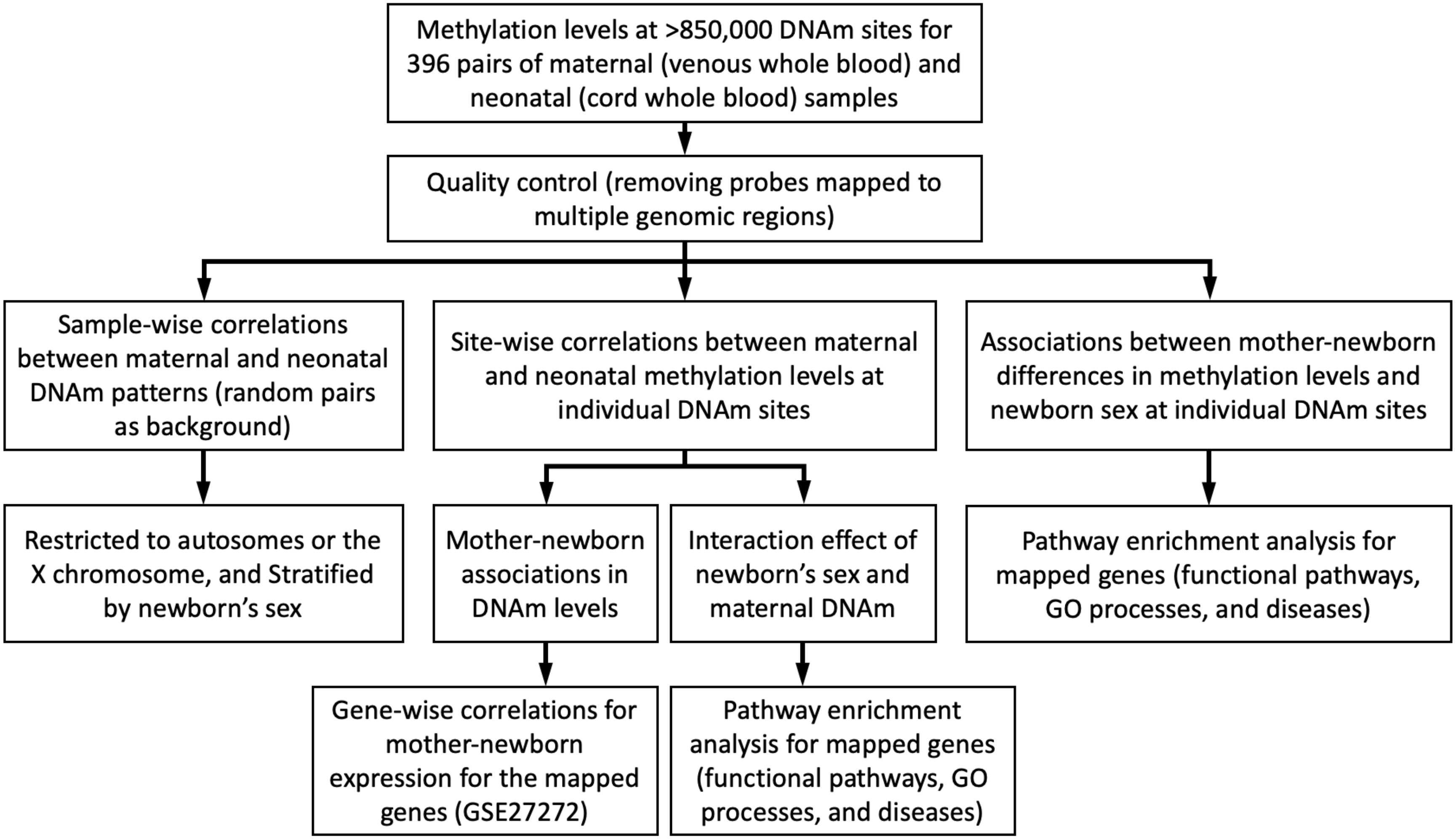

Supplement: Supplementary file 1 — Additional file 1. Supplementary Figures. [file 13148_2023_1442_MOESM1_ESM.pdf]
